# Stringent *in-silico* identification of putative G-protein-coupled receptors (GPCRs) of the entomopathogenic nematode *Heterorhabditis bacteriophora*

**DOI:** 10.2478/jofnem-2023-0038

**Published:** 2023-11-23

**Authors:** Artha Kundu, Nisha Jaiswal, Uma Rao, Vishal Singh Somvanshi

**Affiliations:** Division of Nematology, ICAR-Indian Agricultural Research Institute, New Delhi-12, India

**Keywords:** Bioinformatics, Chemoreception, Entomopathogenic nematodes, NemChRs, Parasites

## Abstract

The infective juveniles (IJs) of entomopathogenic nematode (EPN) *Heterorhabditis bacteriophora* find and infect their host insects in heterogeneous soil ecosystems by sensing a universal host cue (CO_2_) or insect/plant-derived odorants, which bind to various sensory receptors, including G protein-coupled receptors (GPCRs). Nematode chemosensory GPCRs (NemChRs) bind to a diverse set of ligands, including odor molecules. However, there is a lack of information on the NemChRs in EPNs. Here we identified 21 GPCRs in the *H. bacteriophora* genome sequence in a triphasic manner, combining various transmembrane detectors and GPCR predictors based on different algorithms, and considering inherent properties of GPCRs. The pipeline was validated by reciprocal BLAST, InterProscan, GPCR-CA, and NCBI CDD search. Functional classification of predicted GPCRs using Pfam revealed the presence of four NemChRs. Additionally, GPCRs were classified into various families based on the reciprocal BLAST approach into a frizzled type, a secretin type, and 19 rhodopsin types of GPCRs. Gi/o is the most abundant kind of G-protein, having a coupling specificity to all the fetched GPCRs. As the 21 GPCRs identified are expected to play a crucial role in the host-seeking behavior, these might be targeted to develop novel insect-pest management strategies by tweaking EPN IJ behavior, or to design novel anthelminthic drugs. Our new and stringent GPCR detection pipeline may also be used to identify GPCRs from the genome sequence of other organisms.

Nematodes or roundworms are the most abundant metazoans on earth and are found in all conceivable habitats (Blumenthal and Davis, 2004; Bardgett and van der Putten, 2014). They are commonly classified as marine or terrestrial, free-living or animal/human/plant-parasitic (Lambshead, 2004). In order to perceive the environment and seek their hosts in soil or other habitats, nematodes depend on a very well-developed chemosensory mechanism (Curtis, 2008; Reynolds et al., 2011). The nematode *Caenorhabditis elegans* is a well-known model organism and has tremendously contributed to the advancement in understanding of biology (Riddle et al., 1997). Free-living nematodes such as *C. elegans* and most other parasitic nematodes use their anterior and posterior chemosensory organs (known as amphids and phasmids, respectively), to orient themselves in response to chemosensory cues such as host exudates, food signals, sex pheromones, and other small molecules (Shivakumara et al., 2019). Entomopathogenic nematodes (EPNs) are a special class of nematodes that are pathogenic to insects, and are used worldwide for managing insect pests of crops (Koppenhöfer and Fuzy, 2006). Like other nematodes, EPNs too depend upon chemoreception, olfaction, hygrosensation, thermoreception, and gustation to sense and understand their environment and to identify hosts, noxious compounds, and sexual partners, and to undertake alternative developmental pathways (Perry, 1996; Krieger and Breer, 1999; Prasad and Reed, 1999; McGaughran et al., 2013; Shivakumara et al., 2018). The EPN infective juveniles (IJs) use universal host cue CO_2_, and host- and insect-damaged plant-derived odorants to locate their insect hosts in the heterogeneous soil ecosystem (Hallem et al., 2011; Dillman et al., 2012). The EPN *H. bacteriophora* is a successful biocontrol agent for several insect pests of crops (Poinar, 1975), and its small size, short lifespan, ease of culturing, hermaphroditism and close evolutionary relationship with *C. elegans* and other mammalian parasitic nematodes makes it an ideal model for study of insect pathogenesis and other biological studies (O’Halloran and Burnell, 2003; Hallem et al., 2007; Ratnappan et al., 2016).

Olfaction, as a part of chemoreception, uses water-soluble volatile odorants or gaseous chemical cues which bind to various sensory receptors, including G protein-coupled receptors (GPCRs). GPCRs, are also known as seven-transmembrane (7-TM) domain receptors, are the largest family and most diverse group of membrane receptors in eukaryotes including EPNs (Elrod and Chou, 2002; Audebrand et al., 2020). Most GPCRs are 200–1000 amino acids long (Bresso et al., 2019). They are found within the lipid-protein bilayer of the cell membrane and are responsible for regulating communication between the cells and its surroundings (Fig. 1) (Schiöth and Fredriksson, 2005; Sonnabend et al., 2017; Insel et al., 2019). In addition to that, it is suggested that they are present in the cell organelles, and inside the nucleus. There are hundreds of different GPCRs which can bind to a diverse set of ligands, including peptide hormones, neurotransmitters, neuropeptides, biogenic amines, amino acids, ions, lipid-derived mediators, peptides, proteins, and odor molecules (Gether, 2000; Schiöth and Fredriksson, 2005; Hilger et al., 2018; Sriram and Insel 2018) and they are responsible for vision, olfaction, taste, and more (Rosenbaum et al., 2009). Nematode chemosensory GPCRs (NemChRs) are unique to nematodes, and are involved in the detection and reception of odor molecules, as their ligand binding sites are located on cell surfaces and are accessible to sensory molecules (Robertson, 2006; Krishnan et al., 2014). This sensory perception is processed downstream through different signalling pathways leading to modulation of nematode behavior (Bargmann et al., 1993; Bargmann, 2006). Silencing of these downstream effectors inhibits nematode response, activation, and movement towards host-emitted cues (Gang et al., 2020; Wheeler et al., 2020). The GPCRs are involved in controlling the movement of EPNs towards (or within) their host (Bresso et al., 2019). Recently, Bernot et al. (2020) and Wheeler et al. (2020) have reported the involvement of NemChRs in host-seeking behaviour.

**Figure 1:**
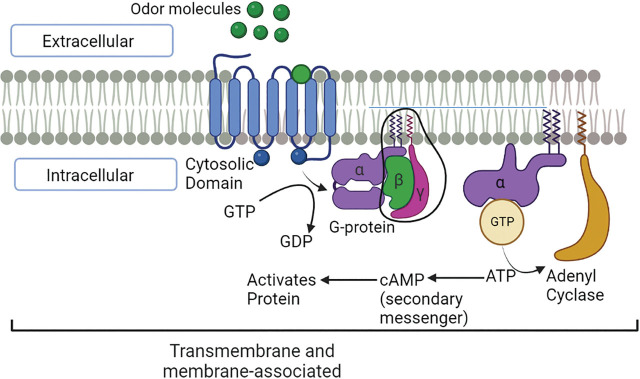
GPCRs are found within the lipid-protein bilayer of the cell membrane and are responsible for regulating communication between cells and its surroundings (Insel et al., 2019; Schiöth & Fredriksson, 2005). GPCR becomes activated by extracellular signal, like ligand binding, resulting in an exchange of guanosine diphosphate (GDP) with guanosine triphosphate (GTP) on the Gα subunit, and a subsequent dissociation of the heterotrimer into Gα and a βγ subunit dimer. Gα and the βγ dimer then proceed to initiate downstream signaling cascades (Davies et al., 2007; Jones, 2002; Morris & Malbon, 1999).

Genome sequencing projects have made it possible for researchers to identify genes of interest in their favorite organisms. *C. elegans* was the first animal genome to be sequenced, and identification of ca. 1000 GPCRs in the *C. elegans* genome (Bargmann, 1998) opened a new paradigm for understanding its chemotactic behavior and related physio-biochemical reactions (Robertson, 2006). However, predicting GPCRs in a genome has not been easy because of the complexity of these genes, and the inability of simple sequence analogy to predict a GPCR (Bockaert and Pin, 1999; Fredriksson et al., 2003; Wistrand et al., 2006; Suwa, 2014). Several tools have been developed for prediction of GPCRs from genome sequences such as GPCRHMM, GPCRPred, GPCRPipe, GPCR-Pen, and so on; however, all of them use different algorithms and databases in the background, resulting in inconsistent outcomes (Bhasin and Raghava, 2004; Wistrand et al., 2006; Theodoropoulou et al., 2013; Begum et al., 2020). For example, even if all the tools are trained on the human genome database, they identify different sequences as GPCRs, thus adding to the confusion (Takeda et al., 2002; Bjarnadottir et al., 2006). Therefore, researchers working on GPCRs are always seeking simpler means for stringent identification of GPCRs in a genome sequence. The genome of entomopathogenic *H. bacteriophora* was sequenced in 2013 and a total of 82 GPCRs were predicted in the genome of *Heterorhabditis bacteriophora* (Bai et al., 2013). In this study, we investigated the proteomic dataset of *H. bacteriophora* (Bai et al., 2013) and its improved annotated version published in 2018 (McLean et al., 2018) to identify putative GPCRs. We designed a pipeline for this purpose by combining several independent trans-membrane (TM) finders and GPCR predictors. We also investigated putative GPCRs in the better annotated genome of model organism *C. elegans* to test the validity of our method. Further, we predicted the functions of the identified GPCRs *in-silico*, and classified them into family and subfamily levels (Attwood and Findlay, 1994). To the best of our knowledge, this is the first pipeline of its kind for stringent identification of GPCRs from a genome sequence, and could be useful for identifying GPCRs from other eukaryotic genome sequence assemblies.

## Materials and Methods

### Identification of putative GPCRs

**Figure 2:**
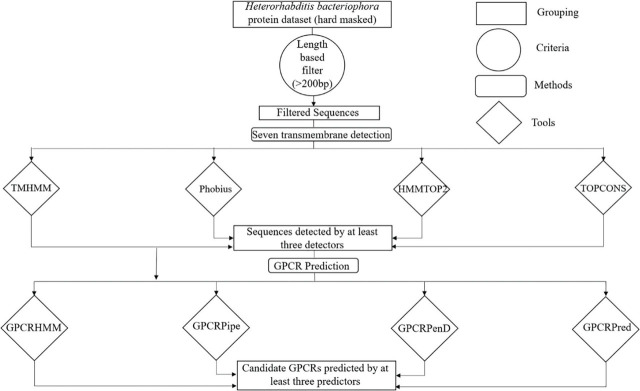
Visual representation of overall strategy to predict and identify novel GPCRs through bioinformatic approach. Groups of sequences are represented by rectangles, tools by rhombuses and criteria by circles.

In order to predict putative GPCRs in *H. bacteriophora* and *C. elegans*, we followed a combination of different bioinformatic approaches, including both motif-based and alignment-free methods, as well as structural similarity–based approaches. The sequence alignment methods were comprised of BLASTX, BLASTP (Altschul et al., 1990), and Pfam (Bateman et al., 2000). The alignment-free method included GPCRPred (Bhasin and Raghava, 2004), whereas TMHMM (Krogh et al., 2001), GPCRHMM (Wistrand et al., 2006), and GPCRPipe (Theodoropoulou et al., 2013) come under machine learning or statistical algorithm-based methods. The entire pipeline for GPCR identification was composed of three key steps, as illustrated in Fig. 2.

### Length-based screening and transmembrane detection

The initial step was to filter the large number of protein sequences based on their length. The proteomic dataset (Bai et al., 2013; Mclean et al., 2018) of *H*. *bacteriophora* and *C. elegans* (*C. elegans* Sequencing Consortium, 1998) containing 21,699, 15,218, and 28,447 predicted protein sequences, respectively, were filtered based on the length of sequences to discard proteins with less than 200 amino acid residues. These sequences were then checked for the number of transmembrane helices. Four different transmembrane detectors were used for this purpose: (1) TMHMM 2.0 (https://services.healthtech.dtu.dk/service.php?TMHMM-2.0), a membrane protein topology prediction method based on hidden Markov model (HMM) (Krogh et al., 2001); (2) Phobius (https://phobius.sbc.su.se/), an HMM-based signal peptide predictor that detects transmembrane protein topology also (Käll et al., 2004; Käll et al., 2007); (3) HMMTOP2 (http://www.enzim.hu/hmmtop/), an automatic server for predicting transmembrane helices and topology of proteins based on HMM (Tusnády and Simon, 1998; Tusnády and Simon, 2001); and (4) TOPCONS (https://topcons.cbr.su.se/), a membrane protein topology prediction tool based on a complex algorithm (Tsirigos et al., 2015). TMHMM2 was run using the “one line per protein” option. Phobius was run in the Normal prediction mode with the short output format mode selected. HMMTOP2 was run in the advanced mode with the parameters: FASTA format, Single Sequence type, Reliable prediction type, text output, and the results in one line. TOPCONS was operated with default parameters.

### GPCR Prediction

Seven transmembrane sequences, detected by any of the three transmembrane detectors, were then subjected to four different GPCR prediction tools to identify putative GPCRs from those sequences. These were GPCRHMM (https://gpcrhmm.sbc.su.se/index.html), an HMM-based GPCR prediction tool (Wistrand et al., 2006); GPCRPipe (http://aias.biol.uoa.gr/GPCRpipe/search.php), which detects GPCRs using pHMM library and HMM for GPCRs (Theodoropoulou et al., 2013); GPCR Prediction Ensemble (GPCR-Pen) (https://gpcr.utep.edu/), which determines GPCR based on four different algorithms (Begum et al., 2020); and GPCRPred (https://webs.iiitd.edu.in/raghava/gpcrpred/), a support vector machine (SVM) based approach (Bhasin and Raghava, 2004). In case of GPCRPipe, it detects GPCRs in two consecutive steps. In the first step, it uses Hidden Markov Model and then a library of 39 Pfam profile HMMs (specific to different families of GPCR). Analyses using GPCRHMM were performed with the local scoring option turned on. We used the GPCRPipe “AND” method as it allows prediction of GPCR only when it is confirmed by two methods. This selection resulted in a reduced number of GPCRs and a limited number of false positives. GPCRPred was run in its webserver without any manipulation in default parameters. While using GPCR-Pen, we restricted the prediction algorithms to Pfam and BLAST to get results only based on sequence similarity, and we fetched those sequences which were confirmed by both of these methods.

The outcomes of all the bioinformatic tools were then compiled and visualized using the Venny 2.1 software (https://bioinfogp.cnb.csic.es/tools/venny/). Venn diagrams were created to compare and to reflect the number of seven transmembrane sequences and predicted GPCRs made by different combinations of tools.

### Pipeline validation and family-wide classification

Apart from predicting GPCR, GPCRPred was also used to know the families and subfamilies of GPCRs based on the dipeptide composition of proteins. It categorizes GPCRs into class A (rhodopsin-like), B (secretin and adhesion), C (metabotropic glutamate), D (fungal pheromone receptors), E (cAMP receptors), or F (frizzled), as per International Union of Basic and Clinical Pharmacology (IUPHAR) nomenclature (http://www.guidetopharmacology.org/nomenclature.jsp; Attwood and Findlay, 1994; Kolakowski, 1994; Sadowski and Parish, 2003).

All GPCR candidates predicted by the pipeline were analyzed by another alignment-free method, GPCR-CA (http://218.65.61.89:8080/bioinfo/GPCR-CA), whose algorithm is based on cellular automation (CA) (Xiao et al., 2009). GPCR-CA is a bilayer predictor: the first layer identifies a query protein as GPCR or non-GPCR; if it is a GPCR, then a second layer classifies it into an A-F classification system, based on sequence homology and functional similarity (Attwood and Findlay, 1994).

In order to validate the pipeline, the putative GPCRs were then checked for the presence of an extracellular N terminus and an intracellular C terminus using HMMTOP2 and TMHMM2.

We screened all these GPCRs through Pfam (http://pfam.xfam.org/) (Punta et al., 2012), a database of protein families and an HMM-based (Eddy, 1998) protein classifier to get a complete and accurate classification of protein families and domains. Apart from this, InterPro (https://www.ebi.ac.uk/interpro/) was also used to analyze GPCR sequences functionally and to classify them into families, and ultimately to predict the presence of signature domains and important sites. We revalidated all these analyses through conserved domain analysis by NCBI CDD search (https://www.ncbi.nlm.nih.gov/Structure/cdd/wrpsb.cgi).

Putative *H. bacteriophora* GPCRs were classified into GPCR families according to the families to which their orthologous proteins in *C. elegans* and *Ancylostoma caninum* were assigned. For this purpose, orthology was detected between these resultant sequences and other organisms with the widely used reciprocal BLAST approach in WormBase Parasite (https://parasite.wormbase.org/index.html). This is done by using each GPCR sequence detected from *H. bacteriophora* as a query to the *Caenorhabditis elegans* proteome and selecting the highest scoring *C. elegans* protein sequence. This *C. elegans* sequence was then used as the query in a BLAST search against the proteome of *H. bacteriophora*. If the *C. elegans* sequence reciprocally identified the original query sequence as the highest scoring hit with the BLAST, these two sequences were then considered as an orthologous pair, which implies that they may possess similar functions and biological roles and fall into the same GPCR subfamily. *H. bacteriophora* GPCRs were assigned to families and subfamilies based on their orthologous GPCRs in other model organisms, such as *C. elegans* and *A. caninum*. In all BLAST comparisons, we used the BLOSUM62 scoring matrix and a cut-off threshold of 1e-1. Additionally, motif prediction of identified GPCRs was performed in MEME Suite 5.4.1 (https://meme-suite.org/meme/). Motifs were functionally annotated in HHpred (http://toolkit.tuebingen.mpg.de/hhpred) using default parameters. Functional motifs were identified in the GPCR sequences using the MOTIF search tool (https://www.genome.jp/tools/motif/).

PRED-COUPLE2 (http://athina.biol.uoa.gr/bioinformatics/PRED-COUPLE2/) (Sgourakis et al., 2005) was used to predict the coupling specificity of GPCRs to the four families of G-proteins, with a stringent cutoff of 0.3 to discriminate between positive and negative predictions. Therefore, results below this limit were not considered as positive predictions and were discarded.

## Results

### Identification of putative GPCRs

**Figure 3:**
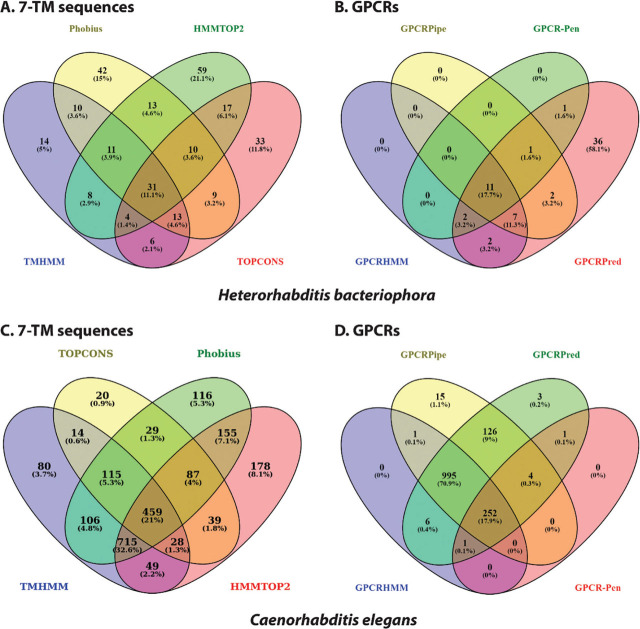
Venn diagram representing numbers of sequences in *Heterorhabditis bacteriophora* genome- A) 7-TM sequences predicted by 4 different tools (TMHMM2, Phobius, HMMTOP2 and TOPCONS); B) GPCRs predicted by 4 different platforms (GPCRHMM, GPCRPipe, GPCRPred and GPCRPenD), and in *Caenorhabditis elegans* genome- C) 7-TM sequences, and D) GPCR sequences identified using the same tools.

Out of the total 21,699 predicted proteins of *H. bacteriophora*, 21 sequences were identified as GPCRs using the pipeline presented in Fig. 2. At the initial stage of identification, 7,613 sequences with more than 200 amino acid residues were short-listed. Four different transmembrane predictors identified varying numbers of sequences containing transmembrane domains. TMHMM2, a widely used transmembrane predictor, identified 97 sequences containing 7-TM helices out of 7,613 sequences, of which 14 7-TM sequences were uniquely identified by TMHMM2 (Fig. 3). Another tool, Phobius, detected 139 7-TM sequences, of which 42 were unique (Fig. 3). In addition, 153 sequences were predicted by HMMTOP2, with 59 unique sequences. TOPCONS could identify only 123 proteins containing 7-TM helices, but only 25% of those were uniquely identified. A total of 69 sequences were predicted to have 7-TM helices by at least three out of four transmembrane predictors used for this study (Fig. 3A, supplementary Table 1).

**Table 1.** The characterization and classification of GPCR sequences identified through a bioinformatics pipeline using various annotation tools and methods, and their probable coupling specificity with different G-proteins

**Query Name**	**Class Identified by GPCR Pred/GPCR-CA class/Reciprocal BLAST**	**GPCRPred Subfamilies**	**InterPro Protein family**	**Pfam Identifier**	**Pfam Details**	**Predicted G-proteins showing coupling specificity**
Hba_04160	Class A Rhodopsin like	Peptide	G protein-coupled receptor, rhodopsin-like (IPR000276)	7tm_1		Gi/o, Gs
Hba_07805	Class A Rhodopsin like	Peptide	G protein-coupled receptor, rhodopsin-like (IPR000276)	7TM_GPCR_Srw	Serpentine type 7TM GPCR chemoreceptor Srw	*Gi/o, Gq/11*
			7TM GPCR, serpentine receptor class w (Srw) (IPR019427)			
Hba_10668	Class A Rhodopsin like	Amine	G protein-coupled receptor, rhodopsin-like (IPR000276)	7tm_1	7 transmembrane receptor (rhodopsin family)	Gi/o, Gs
Hba_12209	Class A Rhodopsin like	Peptide	G protein-coupled receptor, rhodopsin-like (IPR000276)	7tm_1	7 transmembrane receptor (rhodopsin family)	*Gi/o, Gq/11, G12/13*
Hba_14891	Class A Rhodopsin like	Peptide	G protein-coupled receptor, rhodopsin-like (IPR000276)	7tm_1	7 transmembrane receptor (rhodopsin family)	*Gi/o*
Hba_18427	Class A Rhodopsin like	Peptide	7TM GPCR, serpentine receptor class w (Srw) (IPR019427)	7TM_GPCR_Srw	Serpentine type 7TM GPCR chemoreceptor Srw	*Gi/o, Gs, Gq/11*
Hba_18743	Class A Rhodopsin like	Peptide	G protein-coupled receptor, rhodopsin-like (IPR000276)	7TM_GPCR_Srsx	Serpentine type 7TM GPCR chemoreceptor Srsx	*Gq/11, Gi/o, Gs*
			Serpentine type 7TM GPCR chemoreceptor Srsx (IPR019424)			
Hba_18878	Class A Rhodopsin like	Peptide	G protein-coupled receptor, rhodopsin-like (IPR000276)	7tm_1	7 transmembrane receptor (rhodopsin family)	*Gi/o, Gq/11, Gs, G12/13*
			G-protein coupled receptor Aex-2 (IPR039952)			
Hba_18906	Class A Rhodopsin like	Lysospingolipid	None	GpcrRhopsn4	Rhodopsin-like GPCR transmembrane domain	*Gi/o, Gq/11*
Hba_19080	Frizzled	Peptide	Frizzled/secreted frizzled-related protein (IPR015526)	Frizzled	Frizzled/Smoothened family membrane region	*Gi/o, Gq/11, G12/13, Gs*
Hba_19161	Class A Rhodopsin like	Peptide	G protein-coupled receptor, rhodopsin-like (IPR000276)	7tm_1	7 transmembrane receptor (rhodopsin family)	*Gi/o*
Hba_20566	Secretin-like	Rhodopsin	GPCR, family 2, secretin-like (IPR000832)	7tm_2	7 transmembrane receptor (Secretin family)	*Gi/o*
Hba_03545	Class A Rhodopsin like	Peptide	Putative G protein-coupled receptor, Chromadorea (IPR040435)			*Gi/o, Gq/11*
Hba_20096	Class A Rhodopsin like	Peptide	G protein-coupled receptor, rhodopsin-like (IPR000276)	7tm_1		*No Match Found*
Hba_18203	Class A Rhodopsin like	Peptide	G protein-coupled receptor, rhodopsin-like (IPR000276)	7tm_1	7 transmembrane receptor (rhodopsin family)	*Gs, Gi/o*
Hba_12258	Class A Rhodopsin like	Peptide	G protein-coupled receptor, rhodopsin-like (IPR000276)	7tm_1	7 transmembrane receptor (rhodopsin family)	*Gq/11, Gs, Gi/o*
			G-protein coupled receptor Aex-2 (IPR039952)			
Hba_17528	Class A Rhodopsin like / Class D Fungal pheromone like	Peptide	None	7TM_GPCR_Srx	Serpentine type 7TM GPCR chemoreceptor Srx	*Gi/o*
Hba_14446	Class A Rhodopsin like	Amine	G protein-coupled receptor, rhodopsin-like (IPR000276)	7tm_1	7 transmembrane receptor (rhodopsin family)	*Gi/o, Gq/11, G12/13*
Hba_09978	Class A Rhodopsin like	Peptide	G protein-coupled receptor, rhodopsin-like (IPR000276)	7tm_1	7 transmembrane receptor (rhodopsin family)	*Gi/o, Gq/11*
Hba_08130	Class A Rhodopsin like	Peptide	G protein-coupled receptor, rhodopsin-like (IPR000276)	7tm_1	7 transmembrane receptor (rhodopsin family)	*Gi/o, Gq/11, Gs*
Hba_13948	Class A Rhodopsin like	Amine	G protein-coupled receptor, rhodopsin-like (IPR000276)	7TM_GPCR_Srx		*Gi/o, Gq/11, G12/13, Gs*

The common 69 heptathetical transmembrane sequences were passed through four GPCR prediction tools to identify the GPCRs among these sequences. GPCRHMM, GPCRPipe, GPCR-Pen, and GPCRPred identified 22, 21, 15, and 62 sequences as GPCRs, respectively (Fig. 3B). Interestingly, GPCRHMM, GPCRPipe, and GPCR-Pen did not identify any unique GPCR sequence, but GPCRPred identified 36 unique GPCRs not predicted by any other tool. In summary, 21 sequences were considered as putative GPCRs as they were identified by at least three of the four tools used for GPCR prediction, and further confirmed by detailed analysis of the sequences (Fig. 3B, Tables 1–3, supplementary Tables 2 – 4).

**Table 2.** The Gene Ontology annotations of identified GPCR sequences of *H. bacteriophora*.

**Sequence Name**	**Gene Ontology (GO) annotation categories**		

	**Biological function**	**Molecular function**	**Cellular function**
Hba_04160	G protein-coupled receptor signaling pathway (GO:0007186)	G protein-coupled receptor activity (GO:0004930)	Integral component of membrane (GO:0016021)
Hba_07805	G protein-coupled receptor signaling pathway (GO:0007186)	G protein-coupled receptor activity (GO:0004930)	Integral component of membrane (GO:0016021)
		G protein-coupled peptide receptor activity (GO:0008528)	
Hba_10668	G protein-coupled receptor signaling pathway (GO:0007186)	G protein-coupled receptor activity (GO:0004930)	Integral component of membrane (GO:0016021)
Hba_12209	G protein-coupled receptor signaling pathway (GO:0007186)	G protein-coupled receptor activity (GO:0004930)	Integral component of membrane (GO:0016021)
Hba_14891	G protein-coupled receptor signaling pathway (GO:0007186)	G protein-coupled receptor activity (GO:0004930)	Integral component of membrane (GO:0016021)
Hba_18427	G protein-coupled receptor signaling pathway (GO:0007186)	G protein-coupled receptor activity (GO:0004930)	Integral component of membrane (GO:0016021)
		G protein-coupled peptide receptor activity (GO:0008528)	
Hba_18743	G protein-coupled receptor signaling pathway (GO:0007186)	G protein-coupled receptor activity (GO:0004930)	Integral component of membrane (GO:0016021)
Hba_18878	neuropeptide signaling pathway (GO:0007218)	Neuropeptide receptor activity (GO:0008188)	Integral component of membrane (GO:0016021)
	G protein-coupled receptor signaling pathway (GO:0007186)	G protein-coupled receptor activity (GO:0004930)	
Hba_18906	G protein-coupled receptor signaling pathway (GO:0007186)	None	None
	response to pheromone (GO:0019236)		
Hba_19080	Cell surface receptor signaling pathway (GO:0007166)	Protein binding (GO:0005515)	Membrane (GO:0016020)
		transmembrane signaling receptor activity (GO:0004888)	integral component of membrane (GO:0016021)
Hba_19161	G protein-coupled receptor signaling pathway (GO:0007186)	G protein-coupled receptor activity (GO:0004930)	Integral component of membrane (GO:0016021)
Hba_20566	G protein-coupled receptor signaling pathway (GO:0007186)	G protein-coupled receptor activity (GO:0004930)	Integral component of membrane (GO:0016021)
	cell surface receptor signaling pathway (GO:0007166)	transmembrane signaling receptor activity (GO:0004888)	
Hba_03545	None	None	Integral component of membrane (GO:0016021)
Hba_20096	G protein-coupled receptor signaling pathway (GO:0007186)	G protein-coupled receptor activity (GO:0004930)	Integral component of membrane (GO:0016021)
Hba_18203	G protein-coupled receptor signaling pathway (GO:0007186)	G protein-coupled receptor activity (GO:0004930)	Integral component of membrane (GO:0016021)
Hba_12258	Neuropeptide signaling pathway (GO:0007218)	Neuropeptide receptor activity (GO:0008188)	Integral component of membrane (GO:0016021)
	G protein-coupled receptor signaling pathway (GO:0007186)	G protein-coupled receptor activity (GO:0004930)	
Hba_17528	None	None	Integral component of membrane (GO:0016021)
Hba_09978	G protein-coupled receptor signaling pathway (GO:0007186)	G protein-coupled receptor activity (GO:0004930)	Integral component of membrane (GO:0016021)
Hba_14446	G protein-coupled receptor signaling pathway (GO:0007186)	G protein-coupled receptor activity (GO:0004930)	Integral component of membrane (GO:0016021)
Hba_13948	G protein-coupled receptor signaling pathway (GO:0007186)	G protein-coupled receptor activity (GO:0004930)	Integral component of membrane (GO:0016021)
Hba_08130	G protein-coupled receptor signaling pathway (GO:0007186)	G protein-coupled receptor activity (GO:0004930)	Integral component of membrane (GO:0016021)

**Table 3.** Similarity of motifs identified by MEME analysis in GPCRs with the known protein domains as analyzed by HHPred

**Motif**	**Hit (PDB id)**	**Probability**	**E-value**	**SS**	**Target Length**
**Motif 1**	Serpentine Receptor, class V [*Caenorhabditis elegans*]	98.28	1.1e-6	1.9	219
	Human Gonadotropin-releasing hormone Receptor (GnRHR) related [*Caenorhabditis elegans*]	98	9.6e-7	−1.4	257
	BILF1; Membrane protein, viral GPCR, class A-like GPCR, Epstein-Barr virus; HET: Y01{*Homo sapiens*}	97.98	5.4e-8	−6.3	231
**Motif 2**	Type-1 angiotensin II receptor, Soluble cytochrome b562 BRIL fusion protein; GPCR, MEMBRANE PROTEIN; HET: CLR, OLC, NAG	98.17	7.9e-7	0.1	425
	Proteinase-activated receptor 2, soluble cytochrome b562, membrane protein, GPCR, 7TM	97.92	4.4e-6	0.1	437
	Neurotensin receptor type 1, lysozyme chimera; G-protein coupled receptor, neurotensin receptor, G-protein, signaling protein	97.87	0.7e-6	0.4	510
**Motif 3**	Cytochrome c1, heme protein, mitochondrial; cytochrome bc1, Membrane protein, heme protein, rieske iron sulfur protein	68.8	10	2.1	241
	Lysophosphatidic acid receptor 6a, Endolysin, Lysophosphatidic acid receptor 6a; alpha helical, membrane protein; HET: OLC	66.66	10	2.1	241
	Neuropeptide Receptor family [*Caenorhabditis elegans*]	60.14	1.6	−1.5	477
**Motif 4**	Zinc finger protein sdc-1 [*Caenorhabditis elegans*]	27.67	58	0.9	1201
	Uncharacterized protein CELE_ZC204.13 [*Caenorhabditis elegans*]	20.25	85	0.5	156
**Motif 5**	Uncharacterized protein CELE_F56H11.2 [*Caenorhabditis elegans*]	27.34	48	0.3	129
	U4/U6 small nuclear ribonucleoprotein PRP3; spliceosome, assembly, pre-B complex, U1 snRNP, splicing; HET: GTP; 3.4A	27.2	48	0.3	469
	Uncharacterized protein CELE_C37C3.12 [*Caenorhabditis elegans*]	23.12	63	0.3	172
**Motif 6**	Mitochondrial ATP synthase subunit ASA6; mitochondrial ATP synthase dimer flexible coupling cryoEM, proton transport	46.72	12	0	151
	Ubiquitin carboxyl-terminal hydrolase MINDY-1; hydrolase, cysteine protease, isopeptidase and ubiquitin binding; 2.16A	24.2	63	0.4	289
	Uncharacterized protein YdhK; PF07563 family, DUF1541	21.94	56	−0.1	166
**Motif 7**	Uncharacterized protein CELE_Y46G5A.23 [*Caenorhabditis elegans*]	34.58	38	0.8	108
	Uncharacterized protein CELE_F26F2.3 [*Caenorhabditis elegans*]	27.97	55	0.7	283
	Pandonodin; Lasso peptide, RiPPs, unknown function; NMR {*Pandoraea norimbergensis*}	24.59	130	1.5	33
**Motif 8**	Uncharacterized protein CELE_K04C2.5 [*Caenorhabditis elegans*]	77.76	2.1	0.6	108
	Uncharacterized protein CELE_Y71F9B.1 [*Caenorhabditis elegans*]	52	5.6	−0.7	139
	Early E3 18.5 kDa glycoprotein; Ad2 E3-19K-HLA-A2 complex, unique tertiary structure, Adenovirus E3-19K, Immune evasion	42.68	24	0.6	100
**Motif 9**	Signal recognition particle subunit SRP72 [*Caenorhabditis elegans*]	43.18	17	0.1	635
	Aquaporin or aquaglyceroporin related [*Caenorhabditis elegans*]	27.95	38	0	63
	RNA polymerase I-specific transcription initiation factor RRN6; RNA Polymerase I, Pre-initiation complex	26.05	41	−0.1	894
**Motif 10**	Intracellular growth locus, subunit C; *Francisella tularensis*, cell invasion; HET: MSE	40.56	26	0.6	211
	Maternally affected uncoordination [*Caenorhabditis elegans*]	38.96	37	1	258
	Uncharacterized protein CELE_F59H6.15 [*Caenorhabditis elegans*]	36.51	7.9	−1.7	104

Using the same pipeline, 27 GPCR sequences were identified from the proteomic dataset of *H. bacteriophora*, published by McLean and coworkers in 2018. Out of these sequences, 13 were found to be similar, with the GPCRs fetched from the earlier version of annotation, published in 2013 (Table 4).

**Table 4:** Similarity of GPCRs identified from two different versions of annotations of *H. bacteriophora*

**GPCRs fetched from recent annotation (Mclean et al., 2018)**	**Similar GPCRs fetched from previous annotation (Bai et al., 2013)**
g973.t1	No match
g2254.t1	Hba_12209, Hba_14891, Hba_14446
g4175.t1	Hba_08130
g4474.t1	No match
g5555.t1	Hba_14891
g5582.t1	No match
g5664.t1	Hba_10668
g6690.t1	Hba_09978
g7127.t1	Hba_10668
g8593.t1	Hba_10668, Hba_12209
g10582.t1	Hba_10668, Hba_12209, Hba_14446
g11631.t1	Hba_10668
g7125.t1	No match
g1348.t1	No match
g3739.t1	Hba_19080
g6192.t1	No match
g13587.t1	Hba_19080
g13965.t1	No match
g14268.t1	No match
g337.t1	Hba_18743
g8530.t1	No match
g8941.t1	Hba_17528
g8998.t1	No match
g9082.t1	No match
g10420.t1	No match
g116.t1	No match
g8239.t1	No match

A total 1,252 GPCRs (supplementary Table 5) were identified out of the 28,447 predicted proteins of *C. elegans* using the pipeline proposed in this study (Fig. 2). Initially 21,372 sequences with >200 amino acid residues were screened for 7-TM domains. Out of four different transmembrane predictors, Phobius detected the highest number (1782) of 7-TM sequences, with 116 unique ones. TMHMM2 identified 1,566 sequences containing 7-TM helices, of which 80 sequences were unique. In addition, 1,710 sequences were predicted by HMMTOP2, with the highest number (178) of unique sequences. TOPCONS was able to identify only 791 protein coding genes with 7-TM helices, but <1% of those were unique. A total of 1,404 sequences were predicted to have 7-TM helices by at least three out of four tools (Fig. 3C). The 1,404 resultant sequences were screened using four GPCR predictors. GPCRHMM, GPCRPipe, GPCR-Pen, and GPCRPred identified 1,255, 1,393, 258 and 1,388 sequences as GPCRs, respectively (Fig. 3D). Interestingly, GPCRHMM and GPCR-Pen could not identify any unique GPCR sequence. Finally, 1,252 sequences were considered putative GPCRs as they were identified by at least three of the four GPCR detectors (Fig. 3D, supplementary Table 5).

### Further characterization of predicted GPCRs

All the sequences identified as GPCRs above were validated by the GPCR-CA tool, as it uses Cellular Automaton (CA) images to reveal the features hidden in complex protein sequences. It designated all these GPCRs as Class A Rhodopsin-like GPCR, except Hba_17528, which was identified as Class D Fungal pheromone GPCR (Table 1). Further, the predicted GPCRs were classified based on function and protein family, and four chemosensory GPCRs (Hba_07805, Hba_18427, Hba_18743 and Hba_17528), ten 7-transmembrane receptors under the rhodopsin family, one rhodopsin-like GPCR with transmembrane domain, one frizzled type, and one secretin type GPCR were identified (Table 1, supplementary Table 1). In addition, the reciprocal BLAST was used to find the orthologous sequences of the predicted GPCRs from the proteomic dataset of closely related model organisms. This approach also validated the pipeline and confirmed that all the identified protein sequences were GPCRs. Sequence similarities found a frizzled type (Hba_19080), a secretin type (Hba_20566) and the other 19 as rhodopsin types of GPCR (Table 1). The GPCRPred tool classified all the 21 shortlisted sequences as Class A rhodopsin-like GPCR and further classified them into three different subfamilies: peptide (15 sequences), biogenic amine (3 sequences), and lysospingolipid (1 sequence) (Table 1). A search against InterPro database confirmed the families of all the 21 proteins as G protein-coupled receptors except Hba_17528. All the GPCRs were suggested to be involved in G protein-coupled receptor signalling pathway except Hba_19080, which was annotated to have a role in cell surface receptor signalling. Additionally, Hba_18878 was suggested to be involved in the neuropeptide signalling pathway, and Hba_18906 in pheromone responsiveness. Most of them are rhodopsin-like GPCR, except Hba_19080 (frizzled/secreted, frizzled-related protein) and Hba_20566 (secretin-like). All these GPCRs were found to be integral components of the cell membrane by gene ontology (GO) analysis, except Hba_18906 (Table 2).

Additionally, NCBI conserved domain analysis revealed that four identified proteins were members of the class A seven-transmembrane GPCRs and belonged to FMRFamide (Phe-Met-Arg-Phe)-like receptors and related proteins. Eight of the proteins were rhodopsin receptor-like class A family of the seven-transmembrane GPCR superfamily, which constitutes about 90% of all GPCRs. They include light-sensitive rhodopsin and receptors for biogenic amines, lipids, nucleotides, odorants, peptide hormones, and various other ligands. Six of the proteins were broadly classified under the 7tm_GPCRs superfamily. Among these, Hba_18743 was a serpentine type 7TM GPCR chemoreceptor under the Srsx family, the only family among the various superfamilies of chemoreceptors. Another serpentine type of chemoreceptor GPCR (Hba_17528) was found under the srx family, which is a part of the Srg superfamily of chemoreceptors. Interestingly, Hba_20566 was a pigment-dispersing factor receptor (PDFR), a member of the B1 subfamily of class B seven-transmembrane GPCRs, also referred to as the secretin-like receptor family. Hba_18203 was found to be an FMRFamide receptor and a member of the class A family of seven-transmembrane G protein-coupled receptors. Hba_09978 was a cholecystokinin receptor and came under the class A family of seven-transmembrane GPCRs. This group represents four GPCRs that are members of the RFamide receptor family, including cholecystokinin receptors (CCK-AR and CCK-BR), orexin receptors (OXR), neuropeptide FF receptors (NPFFR), and pyroglutamylated RFamide peptide receptors (QRFPR). Hba_08130 was an amine receptor of the class A family of GPCRs, which include adrenoceptors, 5-HT (serotonin) receptors, muscarinic cholinergic receptors, dopamine receptors, histamine receptors, and trace amine receptors (supplementary Table 2).

**Figure 4:**
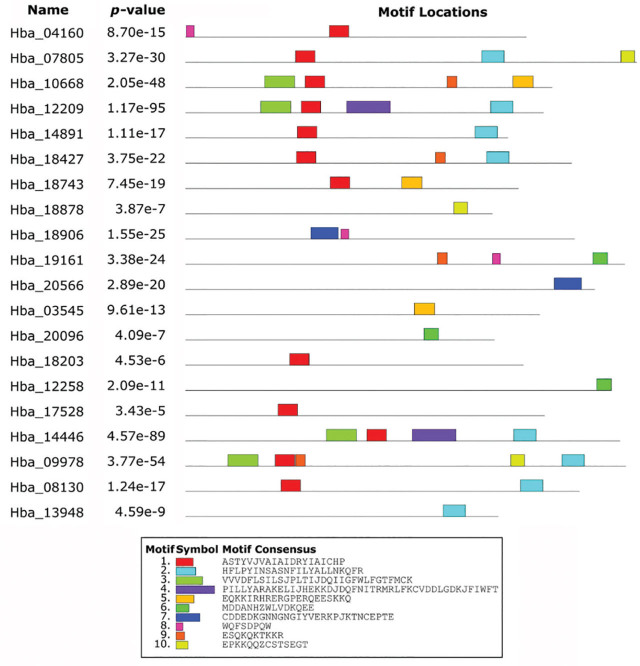
The conserved motif distribution across the 21 GPCRs. Each categorized motif logo generated by MEME is displayed in differentially colored boxes. Legend (in the right-hand side) depicts the protein sequence of corresponding motifs. Motifs were serially numbered according to their frequency of occurrence in MEME bioinformatics tool.

Lastly, in the 21 GPCR sequences, 18 different motifs were identified, which include 7-transmembrane receptor (rhodopsin family), serpentine type 7TM GPCR chemoreceptor Srw, serpentine type 7TM GPCR chemoreceptor Srsx, frizzled/smoothened family membrane region, 7-transmembrane receptor (secretin family), serpentine type 7TM GPCR chemoreceptor Srx, serpentine type 7TM GPCR chemoreceptor Srt, frizzled/smoothened family membrane region etc (Table 3, Fig. 4).

PRED-COUPLE2 analysis showed possible interaction of 21 GPCRs to the different families (Gi/o-, Gq/11-, Gs- and G_12/13_) of G-proteins (Table 1). Among these, Gi/o is the most abundant kind of G-protein, which can bind to all the fetched GPCRs. There are few GPCRs which have coupling specificity only to Gi/o. Additionally, there are promiscuous GPCRs, except Hba_14891, Hba_19161, Hba_20566, and Hba_17528, which can couple to members of more than one G-protein subfamily. Hba_18878, Hba_13948, and Hba_19080 are the only three GPCRs which can interact with all the four types of G-proteins. No coupling specificity is observed with any of the G-proteins; only in the case of Hba_20096. Though Hba_18878 and Hba_19080 have very low sequence similarity, they have coupling ability to members of the same subfamily of G-proteins. While Hba_10668 and Hba_14446 belong to the same amine subfamilies of rhodopsin like GPCR, they often couple to members of distinct G-protein subfamilies. Several sequences under the peptide subfamily have considerably high sequence similarity but they differ in coupling specificity with different families of G-proteins, except G_i/o_ (Table 1).

## Discussion

The entomopathogenic nematodes such as *Heterorhabditis* and *Steinernema* depend on chemoreception to locate and infect their hosts in soil, and are used as biocontrol agents for insect pests of crops (O’Halloran and Burnell, 2003; Hallem et al., 2007; Hallem et al., 2011). In order to perform molecular investigations on chemosensory GPCRs in *Heterorhabditis* nematodes, identification of the GPCRs is the first and most crucial step. We developed a highly stringent bioinformatic pipeline to identify 21 GPCRs in *H. bacteriophora*. These 21 sequences are less than 82 GPCRs identified earlier in the genome of *H. bacteriophora* (Bai et al., 2013), which may be attributed to differences in algorithms and analysis tools used. Bai et al., 2013, searched for presence of GPCR domains in protein sequences based on the HMMER algorithm using Pfam with an e-value cut-off of 1e-4. However, the primary prerequisite for a protein to be a GPCR is the presence of seven stretches of 25–35 consecutive amino acid residues (Fredriksson et al., 2003; Kroeze et al., 2003; Schiöth and Fredriksson, 2005; Rosenbaum et al., 2009; Trzaskowski et al., 2012). To become thermodynamically stable, a transmembrane protein should be folded and packed in such a way that the average length of surface loops and helices of proteins remains 19 and 26 (Meruelo et al., 2012). Therefore, the minimum length of a 7-transmembrane domain containing a protein-coding DNA sequence should be 296 bp. Accordingly, the sequence length cutoff of 200 bp was used in this study.

Langeland et al., 2021 created an integrative database of nematode chemoreceptors called NemChR-DB to facilitate the analysis of NemChRs (http://ohalloranlab.net/nemchr-db). In this database, 53 proteins from *H. bacteriophora* were annotated as NemChRs compared to four NemChRs (Hba_07805, Hba_18427, Hba_18743, and Hba_17528) identified in our study. These four proteins are also listed in NemChR-DB; a total of only seven GPCRs are identified by both our study and Langeland et al., 2021. The reason for the mismatch in numbers of GPCRs identified may be because the present study used highly stringent parameters for identifying a protein as GPCR. Although Langeland et al., 2021 state that 7-TM helices are considered as ideal characteristics for any GPCR (http://ohalloranlab.net/nemchr-db/methods.html), they have also included proteins with 5, 6, 8, and 9 TM domains as GPCR in NemChRs. Their approach is inclusive, whereas we followed highly reductionist approach. In our study, in addition to the 7-TM sequences, four additional, and different, tools were used to search for GPCRs, resulting in a smaller set of sequences being identified as GPCRs as compared to the set identified by Langeland et al. Further, to predict NemChRs, Langeland et al., 2021 and Wheeler et al., 2020 used “hmmsearch” to compare proteins against a database of GPCR Pfam hidden Markov models (HMMs). To revalidate our findings, we also screened the 21 GPCRs through Pfam search, and all were confirmed to contain GPCR domains. Bernot et al., 2020 investigated the expression of GPCRs of *Ancylostoma ceylanicum* across various life stages, and GPCRs were identified based on annotations of the sequences resulting from the transcriptomes. It may be suggested that the GPCRs identified using our methodology are accurate and precise due to higher stringency in the filtration criteria as compared to these studies. Our pipeline identified 1,252 *C. elegans* proteins as GPCRs, a number very close to 1,100 and 1,341 GPCRs reported previously (Bargmann, 1998; Thomas and Robertson, 2008), thus validating the correctness of our method.

Further, these filtered sequences were passed through different transmembrane detectors; α-helical transmembrane proteins are the most important class of membrane proteins and constitute almost 20–30% of all the proteins encoded in a genome (Wallin and von Heijne, 1998; Krogh et al., 2001). From the retained sequences, TMHMM2 detected 97 sequences containing seven transmembrane helices while Phobius, HMMTOP2, and TOPCONS identified 139, 153, and 123 sequences with the same property, respectively. There are more than ten *in-silico* programs available at public interface to identify transmembrane domains. A comparative study proved that TMHMM2 and HMMTOP2 were more accurate than other programs and Phobius was reported to perform comparably (Cuthbertson et al., 2005). TMHMM2 is highly specific and sensitive with >99% accuracy in differentiating membrane and soluble proteins. Additionally, it is highly accurate (97.5%) in predicting helical regions of a transmembrane protein (Krogh et al., 2001). Along with TMHMM2, all the three transmembrane detectors are highly precise in predicting helical regions. If we consider only membrane protein detection with no signal peptide, performance of TOPCONS is around 80%. Though it follows the Hidden Markov Model for transmembrane detection and structure prediction, it relies on a Viterbi-like algorithm to score the final topology model (Tsirigos et al., 2015). Phobius, which is based on HMM, models the different sequence regions of a signal peptide and the different regions of a transmembrane protein in a series of interconnected states with an accuracy of 63.6%, while predicting only TM (Käll et al., 2004). Errors may occur in ORF identification or transmembrane prediction by a single bioinformatic tool. Though all the tools use similar kinds of HMM architecture, the methods and pathways involved in the prediction process are quite different. Their accuracies are also different, due to variation in cross-validation methods and datasets used in the background. So the use of four different transmembrane detectors provided accurate prediction and reduced chances of generating any false positives or negatives, thereby strengthening the GPCR detection pipeline. Among the queries, 69 proteins were considered as seven transmembrane sequences, as those were confirmed by at least three out of four transmembrane detectors.

Among these 7-TM sequences, 22 sequences were identified as GPCRs by GPCRHMM, a Hidden Markov Model–based GPCR recognition software which identifies TM topology-related features. It captures the variation in amino acid composition and topological segment lengths between GPCR families. It has a bare minimum error rate of identification of GPCR, in comparison to other HMM–based GPCR predictors, including Pfam. It has shown a higher percentage of selectivity and sensitivity over profile HMMs and generic transmembrane detectors on sets of known GPCRs and non-GPCRs (Wistrand et al., 2006). As GPCRHMM and GPCRPipe use a similar type of algorithm to detect GPCRs, they identified nearly the same numbers of GPCRs. The “AND” method of GPCRPipe has an accuracy of 97% and sensitivity and specificity of around 91% and 100%, respectively. These values are higher than any other GPCR detectors (Theodoropoulou et al., 2013). On the other hand, GPCRPen comprises sequence similarities (BLAST), common sequence motif profiles (Pfam), transmembrane structure (GPCRTm), and dipeptide composition (GPCRPred) (Begum et al., 2020). But it has only predicted 15 GPCR sequences, as we have restricted our search to the first two algorithms. When a completely different GPCR prediction server (GPCRPred) was employed for the same purpose, it recognized a much higher number of sequences (62) as GPCRs, compared to the other three tools. It is a support vector machine based on dipeptide composition (Bhasin and Raghava, 2004). The completely different search algorithm and trained database resulted in a higher number of sequences, which is almost 3–4 times the number of sequences fetched by the remaining three programs. GPCR recognition accuracy of GPCRPred is up to 99.5% using 5-fold cross-validation. All the resultant sequences were reconfirmed by screening through GPCR-CA, which proves that the pipeline is highly stringent, as it depends on a completely different algorithm to predict and classify GPCRs, which was not used at any earlier stage of the pipeline. It utilizes CA images to reveal the features hidden in a bunch of long and complex protein sequences. The gray-level co-occurrence matrix factors extracted from these images are used to represent the samples of proteins through their pseudo amino acid composition. It designated all these GPCRs as Class A rhodopsin-like GPCRs. Likewise, GPCRPred has also classified all the fetched GPCRs as Class A rhodopsin types. GPCRPred can classify GPCRs into five major classes or families with an overall Matthew's correlation coefficient (MCC) and accuracy of 0.81 and 97.5%, respectively (Bhasin and Raghava, 2004). It has been suggested that despite having low sequence similarity and diversified signal molecules, GPCRs involved in chemoreception might have originated from the rhodopsin family of GPCRs (Nordström et al., 2011). This rhodopsin family is the most abundant and diverse among all the GPCR families. They also have a unique signal transduction mechanism (Rosenbaum et al., 2009). Presence of an extracellular N-terminus domain, an intracellular C-terminus domain, and seven serial transmembrane hydrophobic helices joined by intracellular and extracellular loops are typical properties of GPCRs (Brody and Cravchik, 2000; Kroeze et al., 2003; Rosenbaum et al., 2009; Hanlon and Andrew, 2015). Except Hba_18906, all the retrieved sequences exhibited these properties, which strengthens our pipeline and the selection.

The same methodology of mining GPCRs was applied to the *C. elegans* proteomic dataset. Earlier, Troemel and coworkers in 1995 found a large set of GPCRs (~1341) from the *C. elegans* genome (Troemel et al., 1995). Bargmann (1998) reported around 1100 GPCRs in *C. elegans*. Based on manual curation and sequence comparisons, Thomas and Robertson in 2008 identified nearly 1,300 genes encoding GPCRs in this nematode. Most of these are NemChRs, which are believed to be involved in sensing external environment in absence of visual and auditory systems in *C. elegans*. The number of GPCRs predicted by all these studies is similar to 1,252 GPCRs identified in our study in the genome of *C. elegans*, thus confirming the accuracy and preciseness of the pipeline used in the present study.

Involvement of GPCRs in a wide array of physiological and pathological processes (Dryer and Berghard, 1999; Mombaerts, 1999; Schiöth and Fredriksson, 2005; Nei et al., 2008), and the presence of their ligand binding sites on cell surfaces, have made them the most suitable and accessible drug targets, for, for example, angiotensin receptor blockers (ARBs) for hypertension (Ghosh et al., 2015; Odoemelam et al., 2020; Alhosaini et al., 2021). There are several GPCRs with unknown ligand binding properties, known as orphan GPCRs. GPCR-ligand interaction and its downstream effect is dependent on the interaction of the GPCR under study with members of a specific G-protein subfamily. Therefore, predicting coupling specificity of orphan GPCRs to G-protein subfamilies is essential to find potential drug targets through heterologous expression studies (Wess, 1998). However, GPCRs with low sequence similarity may couple to members of the same subfamily of G-proteins, while members of the same GPCR subfamilies often couple to members of distinct G-protein subfamilies (Wong, 2003). As promiscuous GPCRs are found to be coupled with more than one G-protein subfamily, it is evident that coupling is a multidimensional function rather than one-by-one function (Hermans, 2003; Sgourakis et al., 2005).

The NemChRs identified in this study must be functionally validated for their roles in chemoreception in EPN *H. bacteriophora*. NemChRs are generally expressed in amphid sensory neurons (Vidal et al., 2018) and are expected to facilitate host recognition, seeking, and detection (Bernot et al., 2020; Wheeler et al., 2020). GPCRs, like NemChRs, may be responsible for attraction and movement towards a host, and may be manipulated to modulate the nematode behavior in soil, as shown in the case of root-knot nematode *M. incognita* (Bresso et al., 2019). Like NemChRs, these GPCRs may also provide ideal drug targets for nematicides and anthelminthics (Robertson, 2006; Krishnan et al., 2014). Lastly, the bioinformatic pipeline developed in this study may be used to identify GPCRs from the genome of any organism, including nematodes.

## Figures and Tables

**Supplementary Table 1:** List of seven transmembrane sequences (69) fetched from three out of four transmembrane detectors used in this study

>Hba_12006 transcript=Hba_12006 gene=Hba_12006
MFNTSISSMGSNFFADYPNLEEIILRDNSLKRMPGLQMSEEHKMDVEVDLSGNQIGYIGDGRVRGVRARSLRISHNKIKEIAGYAFSGSSFLKLALNGNEELSDLSTDAFKNIIELHHFLLYSAFQNSTFCYCRCRQCSRKAIMNFLRHQEEYGTKKSSGRLSKLNDREKGKFCGLRRITQSASLESNLWAILVRRIYADNRQFETVNDLQSAISKAWNEVDEGVIKNLVNSMTERISRLLIEMYVDDVVEGENLYKNNVKEIHNRICKEREKTERREKRILRQVKNYFLKIKTPSYTHFILYSYWVLRKTIWVVWVLAFVGNILVWIILSLAYERRMRVHYLFMINLSIADLITAIQDVRTADEYYRHAVDWQTSLGCNAGFLAVFASKLSIISMFLIAFEMAYNTRKAFYGHRMNYVTGWGLMVGGWLFSIFMATLPLLGVSSYSATSICLPIRMENVFDRTYIISFLMFNIFAFAGMAVCYAFLICMLKNPRTPSRAEDKDIMLKALLVGTDMLCRFPTLIVGVFAAMGKPLISLSDAKILLVVFFPINAFANPFLYVFCTKVIQRNVKTKAMPVIRRITSTSGVALNSLSNFYHSQPPGNPRHDLSSSPSRLAVTQVTSLNSTPRESNCSSKAVDSLDPSDNTRRSSPRVSFQDDFSTTSRSSSGEYSRRSFGLLKRISALPVSAIPEVSDLSEHSSESHHEHIPKQRRRRFTIYRSLGRYRNESARSAGQDSGRGSLTSIGTTNTYSSKGDQHCC
>Hba_12629 transcript=Hba_12629 gene=Hba_12629
MSRLSARLIFTIFVHGIFSQFGDIMTACFNLLNVPIKQFFHKNMLNKFFKEETFSHNQSTTRGLVLFSYYSVCAASMFFGLMLGFAVLGNLMDHIGRKETGLTTMVVNSGGVIFVLLITPFCTPILLSEDLWFIVPCSCIILATLHLSIAVFFPQSPKQLYIQKHDEDGTRKALEFYLGENCNTKKILNEYEAEKSLESDTFLSLLDVLRDSIYRSTFFLVLICSFVPVFSAVNIKSQYLSSILIGYGLTQAEHSLCWMFAVFGCVFGQIAVNMGLLILSPLMISEICEHKTTAVVSQFTQVVPIMFAVVEVFMFPNLQEIFGASLFFFTPVAV
>Hba_16061 transcript=Hba_16061 gene=Hba_16061
MADGFWITFLGQTIVGASQMFTLGIPPRLAAVWFGPDEVSTACAAGVFGNQLGIAVGFVTPALIVHSGTKDAVAWDLNILFLCSAVLNTLILACIICFFSARPLVPPSLAQLTAQEEKTFDSNFWETKKLMTGKDFLLLFITYGINTGVFYAVSTLLSQMVSFFYKNKTETVGQIGLVIVLAGMLGSVVGGYCLDRFHKFKLTTILVYLFSFLGMLSFTFTINLGSIMIVFGNAFLLGFFMTGYLPIGFEFAAETYPAAEGTTSGLLNASAQVLP
>Hba_20623 transcript=Hba_20623 gene=Hba_20623
MLSRNKLLAEDTSYIKLVLHFGVHLLGVHLFILTQVIGIQCFYPTKINIQKNWQNMQYTRNRPVYCCSVPLLPDITRPNPHLAVAINQKCPVYGTMRVADELLKDASELRRPSASLDSNCRTSNATQLPNEESTMAIPKSVPDVRKFRPFTMNLMTDVSILFADIAGFTKMSSNKSADELVSSILCFFHFSNFHIGMFRINSKWFNLCKPPRFDFLCRTGGLEKISTLGSTPEAHNSFNEKIDQKSPVNGSSSNMKGSRSSGLQLSLQDGNSELNSVGGLDTAISHHHNAASLTRFDTDNKDFDQRLALVIGQGEGGFEKGFWAHHDSLNKWTLKFIEKVRDENSADRHASQQTPLGRHHAKTDIIIEDIPRREDNDDSSQRYSGVFIDILVAALILVVVSAILFMSLSPLPFIFLLYFCIALTTVVAAVGLIESYSSGALLIALPVGVALCVVPLCMFEGCPSVALLSSRLLFSYIMILALFAHCNFSQLGAWPKTLQCIFVGLIHVAVYYCQLNIDNLAEPVEQCVNESRAAFDPSFGSPLFIWEIVLDVGLSIILVGFLNYQFEAAFRMSFFGDVQARRDTERMQVGKIKTIINIQKTFLTQKEVYQIVRDQADWLLNNVIPAHAVESLKTDKYSENHESTAVLFASITNWNDMYEENFEGGREFLRVLNEVIGDFDELLDRPEFCHIEKIKTIGAAYMAASGLNPERKKNMEHPKEHLYQMVEFALAIQHALSVFNEDLLNFDFVCKLGLNIGPITAGIGTTKLYYDIWGDTVNIASRMYSTGVLNRIQVSQHTRDLLLDRYDFEYRDHIEVKGVDGGMDTYLLVGRKEEKYHLQ
>Hba_02250 transcript=Hba_02250 gene=Hba_02250
MAKVEVQIRSCFCCGLSLATIFVALYTLMQRPITVRLDYTLRNSNMKPHVSQFVLFYFPFVDFTIFIFYRYIQVRYMILLVNVFIYVAMIIASILLLIGLSIYNQWLFVPWIIVISIDIIRGLISVLIFIFSYLNLARIATGIFFLGLQFFHVSVNCFQHCLLIFPTKFLTFQISLLMIIIAKFQRIHNKKNGITMDRPVGVLNAASALFLLVLAVFLSCSLYDPRAYLGGTSSTYAYSPETRKADYYPDHQIRQRDPYYSRDVNPGPHDNHGYRY
>Hba_13382 transcript=Hba_13382 gene=Hba_13382
MDLLILFKTLVNVFSVVLHSSLLLIISCTPSLKSGSFDELFFSQNLGDIIAVILRVLTKYIPVLVISNHSGVIEVDRSTFPLATFTNAAEWYSNYVIILSALVFSIDRAVMAMAIRSYSWFCNRVFKCLVILTWLVPLAWVLPMLDKCCKSQYYGGRIELVEMQLHDTDSTYDAIDMVFETLFYVPVLCILNGFSIVMLYSQWNTLQRKRQESKLKKDIQLQLPTICDVHMLCLDRESAHIFPRTHSVDKDFYERMLHRLELELSFSMISVIDQQIDFCAVLFYSLYWASYSRYISVEDTPFLEVYSYFFEFGTILTPYILLFFCSDLRKEFFHRLKPTGILDDDEFTE
>Hba_15897 transcript=Hba_15897 gene=Hba_15897
MSITVVKCILLSIMALVTLVFGLAPIKVLSTLQYGSSGLNAYASLVISLLSCFAGGVFLGVCFLDLMPDALEDEVYRVRAATYLTNGQAELGSVNRKEKETGKSVVKSITLVMALLFHASLEGFAFGQAISSLFFGIIVHKAVVSFSVGMRLVQAHPDRFWLVFILILTIALIAPFGGAIGIIVEGSKINMVTKNIVTCVLLCIALGTFIYLTFMEIIAPEHSNRHSKIAQWCASFLGFAVIAGMMAFGD
>Hba_16327 transcript=Hba_16327 gene=Hba_16327
MPAWQRIIETTLFVPVGLWIVYTTVPLLDCAFSSRPKNYSRYAVLTLYSLIFGAELAFKMISKRTCFLFRLNMYFMPGAFFALAFPILNTRFTRAYRVIAVAHQFLLIPIISKTYSAIASSIIDVFPLQYKIYSNMVHFNELVILVCLIHAVSTDRFVTQLLEHLLTDYNKNVRPVKNASDSLHVKFGANLCRLIDVDEVNQVLTTSLWLEMQWSDRKLVWNPFEWGGVERVHIPSDQIWIPDILLYNNADGEPITIDSLALVDYRGTVLWQPPSIYKSLCPINIEYFPYDSQECHLKFGGWSNDGQTLALEQIPVNIHDRPVKKIDSTGTEFLYLEQGLGLSFYHESAEWDLLRATSSRYAQIYPGCCGQQYYIDIRYNIIRRKAIFFTVMLTIPCMLIANTTPFVFMIPPNEHKMTFSISIFVAFTVFYLVLIELIPPTSLVLPLIGKYLLFTLMMITVSILISVVNINIYRRQAFASEMSPWQRWLFVKTLPKYFRLKRLAIDTSSVESSVTITKGTWVFLDSLTHGEPKLDKKGKLFLKCHDYGFSVSYR
>Hba_16779 transcript=Hba_16779 gene=Hba_16779
MQRVYETLAVCALLIVVILCLLDVVLALLSSSVSLLSITSVNLPLIYSCVSFCGVGLLLVSTPIGFARMFSIVGEMAIIPLRQKSLRWYHHVMLIIKYPIIIISLLMLTGLSLLMVIINSLSLMFGYGLPAYAQYMEVQTRHTLGMPGAIAESITIFYFERYCGILVFYRYVMATSLVGLYSMPFVRTLRPIPGKTSMTAIIINCSLVLVLSSALPVLANTLGITTFDLLGAYSSLEWLSNFRLVLAYNVLFAVTMFCLVSQITNPVRKQIIKRLALKFSITVNFFPLFMLTLLLYYCYNAYKRYKFFRLYELRCRHTRPDELCEKWE
>Hba_19632 transcript=Hba_19632 gene=Hba_19632
MVWGAFSSTGLIDLAFVSTKINSVDYQDVLGNHLVPYLQRFPSVSFTFHQDNATIHASRSTKTWLEDNDVDILDWPSCSSNLNPMEKLRAILVYIGTEFIFYLISPVFLLALRHSPQFGFALSLVTIASAGLNTVKIIQRNFPPTQFLWRQPEMFNPNFIQHHIELYIKPQYRIGPYIVGILLGYYLACYQRQPVRATRSTQFIVLGWMFGIVSGFWALYGLYPALQGWNWPIYHLVYGSIHRDVFAVSMAWLIACHTGIGGIVNKALSAGILLPLSNLCFSVYLFHMIPVVLTYFLVPFPIWYNSKLPIFAHCFVQLLISYFFAIICTMVAEYPALNIERILLAPRPKKTTLKPVPTSDSELQLKPSSPKL
>Hba_20334 transcript=Hba_20334 gene=Hba_20334
MREDWFEIVSTSFALLFGFPSIILCICFTKAVCSGNSSLNTPFFNIFMIAGINDIAVFILNLLSTRLPGTGLFNPTLNSISSGPIPTTIMFLFYLASHAQIYGALIITLNRFTAVAFPTLCLKVDIMVSIFIYNTYNILVYQIWSRISVPCVIMMFVLPIAFCWKIAFHGAVFELYDHPFAEFFYNKPTDQNYISKGVNVTYSIVVVSFINLAFNLGSLGILFHRRKKKQFIKSQKVEFHLYITTLVLFALQILAIQQKSIVTTHVCDDDGGGGDDGDDLPRLQLPKYVKLILKHQTSNKCDLMAIIMASVNYQ
>Hba_20674 transcript=Hba_20674 gene=Hba_20674
MTCAMSVILIMQIQIEKRAVIFGTIGSIPGFIVGSLFIDVYLTSQQKKMLFVSIWSSFAIALFILNVQHGRKTYDIIPNFKPWKASVLIMTGLVGGIFTAFAGSGVDICVFSILTLLFRVTEKTATPSVVLMGINTMIGVYWRAVWEGNISNLALEYAIVSVPIAVTMAPLGSFLGSHLHRQILAIFIYVLEGLAVIGFIITKPAINLMINGAIIVFVAFIFFICISKAGKKLIQNEEALRYQTPESLNDLII
>Hba_20818 transcript=Hba_20818 gene=Hba_20818
MNFVKIYKILIRLHHYTATTCKYNPLSFLPRFLYEQFRRYNNVFFLVIALLQQIPDVSPTGRYTTAVPFLIILSVSAIKEIFEDIIRVGDLLRIENNQLFPADLLLVASSEPQGMAYIETSNLDGETLKIKQALESTSSITSLDKLSKFHAEIDCEPPSRHVNEFSGNLCFMGQQQHFGIDQLLLRGARLKNTSWIFGAIIYTGHDSRLLMNSKTAPLKRQLNICLIFLLVLIRLIRLQSFNTNHFSGTVDIKTSRIIFLFFVLVALALISAAGAEMWRGTNLPQAWYLSFLQMYDINSDSCAVARTSNLNEELGLVKFIMSDKTGTLTRNVMKFKRISVGGTNYGDNENDEFCDESIIQQYKEDPLSEIGVAIKEILTMMVCHTVVPENKDGEILYQSSSPDEGALVRGAANLGFVFHTRQPQKVVVRVLDSDEHLEVLDVVDFTSDRKRMSVIVRDSHGTIKLYTKGADSVIFERLEFRNDKDVETICRNHLEDYASFGYRTLCFAKEIDSVSNSFKLNAVDIQDTYSKWSVDYREASLKIEGRQKALAEVAENIEQNLRLVGATAIEDKLQEFVPETVQALMAANIRVWMLTGDKRETAVNIGLFIATLFFCLNSSVMFFFTFYVFRFAIFSWSGQTIFERWTIGMFNVIFTAWPPVVLGLFDRPVSAQQMMNYPSLYNSFQKRSFSTWQFSLWIGMAIVHSLSLFFLSYAFLEKQVVWENGRTGGWLMLGNCAYTFVVATVCLKAMLECDSWSFPVIFCIGSVALWLLFLFIYAMR
>Hba_20837 transcript=Hba_20837 gene=Hba_20837
MNFTASSPTNRETYDIPVNVCSSLQDLLDSAADKPDLQYRSFSNITVSNVTTTLPMHFSWHDINAHVNEGRRNKIWSCCREPDVEMYKFRKKQVLNSVFGAAHPGKVLAIMGASGAGKTSLLNILAQNTGALEVRGSMKVNGSRVDRMFMKKFSAYVQQDDCFIGSLTVEEHLLFNAKLRMGNRYPEIVQAKRVRQVMRDVGLFTANSVCCFQSVLSRQFQLGLKGSANSLIGTRTRKGLSGGEKKRLAFASEVSVHCLCRCQFQFAFAVLLTSSRIRFKILTSPPILLCDEPTSGLDSFLAFQVVTREPKSSLFGGQVLKKLAEKKGMTILLTIHQPSSQVFELFDRIYLMAEGRVAFCGSQTEAVDFWTQMGNPLPCNFPSDHYISTLAIREKKSRQRQRDAVMAFCDEFARSPTGQAAMVEASSRTGGSRESSENSSSSWESGENYSKTHYASGWCQQLSALHWRNTKTILREPTLLKVQLVQSIIIAILTGIFYLGNTISQEQVNINGSLFQMVTNMAFMFQFSVVNHFCLEMNTFHREHSSGLYRVSTYFLAKNLAELPNYTLSAIIFASILYWMSQMIPLWSAFLFYVMIAILVQNTAISIGSFSSTTCFLENLTFVGAASVADSLITDSFLFLQNINTSLIEGYAFGCIFGNVAVAVAVMPIFVVPLLAFGVDERSVNGTPQTTLKVFAFYAKNPTYESRTWLLRLGFYINQSSLPIYFVPFRFISYFGYAYESLAINEWSHIDSIPGCTHNTNTCYNSGTTVIRSLSFSPENMWINVGIILGMIVLIRFIAFLGLYTRAQLKK
>Hba_21020 transcript=Hba_21020 gene=Hba_21020
MHVLVQLIQNIKQTYNLAVSGVLLAIVGLIGLVGNLLVILVYTHPDQRIHSTSIYLAALASSDFFLILTAMFLFVLEAWRHHNYPLLAWAYVFGAPLVFPIAAVFQTSSVYYCVAAAVDCFIAVVLPSVRQLYCTPKRAKVTCVCIMLLCVIYNIPHFFELEEVACVGNDGLPSTQICPTDIRLDPAYYAIYYTYMYTTFLAIGPLTLLVLLNICVVFTVLTKGTGGALLYNLVTPTRSVSFLLFSFSSYATSQY
>Hba_03545 transcript=Hba_03545 gene=Hba_03545
MGISIYFAMRKFISETNCLMKKFHDSLMDSFAQITRECAQRLEQDEYEEVSLSTILTIAPIFSIISFVVCIVLIVFLIFALIKRRIPSRKYSIVLSRTVADLFTSAFVGAAFLLANLHSASYAILALLYVATFGFVLISFSHLFVIILRQMSVTRPYGFQSTCTMRRICTAVAVTWIMSILYAVSFAPLTTVVLDPSRSKDVCSYGSCQKPMLVIVISFIIAFLIIVFCSYCAVTYKMKKIAHSERMHNEPEVTKKMLKFLVFGGHIALYILISILILIGAGIILHNVWLYNQIRNLMKEKCDVVEYINTLIRLETIAGGAIILWLVRIVFDVFIVFITEYRRLIPCLTTDNLEPLRNSPHSSID
>Hba_04160 transcript=Hba_04160 gene=Hba_04160
MWQGSQPQWLAKPLYGLAAPIIILITLVTNSFVVVVLSNKNLRTPTNHILLSMAITELMTGKEQEGNFNVYIYVGLHILLSANILEVKYFSGLSSCPWFLYYYTFDGLYVDHEEGLPDFWCKAHPYFVHLPTMSVININFCSLYLFHTAAIWLTVYLAIQRYIYVCLPSLVVRFCCPKRTKHIIIYICIVAFVSCVLQQSQVIGVDSWKAGLLCTRALFVHILPCALLIIFTACLFKTVNEADRKRSVHQMLSRQTIGSRCLSSNRRMHATTRMLVVVIGIFLLIEIPVALIFILHLLVVFYGLLDMSDYETINNLLIIRNFLIILTYPLNFAIYFGMSSSFKLQVGIIFL
>Hba_06331 transcript=Hba_06331 gene=Hba_06331
MEKDISEALSQVLHENGFKFTAERVVEDSTTVQVKNPAARKTGEEMEVFFSRTTTLLSQPNSDDEDFGVVLYNNRIISRKMLQYGYIIISLCIYFRSLNFRHGHRPPLPSFAQCFKSIWSLHTETGNWTHLIGCIAFLFLAVWFLTRPDNHIQWQEKVVFSFFFAGAVVCLGLSFAFHTLSCHSINVVRIFCKLDYMGISLLIVGSFIPWIYYGFYCRREPKITYIAMVCVLGFGAIIVSLWDKFSESRYRPFRGVFVGMGCSDNAFHWLLLMAFLYLLGALLYATRTPERFFPGKCDIWFQSHQLFHTCVVIAAFVHYYGISEMAMIRLNGHCPAELSPAHIEL
>Hba_06378 transcript=Hba_06378 gene=Hba_06378
MDNHNMEDAAVREWVSSSYFLYVSVISVSILTLPLLLLSLVSLFKHRKSHYFVCLSSIITGNIVLLDVVQGSAMCKTNAFLVNVSSCFVHWTWVVMYLQRCVCVFFPMRARRNRNLVNGWCAIFGSHLQAVAIAECVLTFFVPFVLTVLTDISVVVLRRPCRPQFILIPADAIAGCDPPSHDGSYLKIVSRKNIDVSYRIFQFLIFCSSIMFFLVVFSSVANFYHLFCFQADAAAYVLYLLQFPLVPIYIYLLKDLDKSLDRRAIRRRMDTSCRLQDPSL
>Hba_07805 transcript=Hba_07805 gene=Hba_07805
MSTNSSNSSAECGLEPHDYLEVKFFLISVIGSLIGIFGLFGNASTALILTRPSMRNPNNLFLTALAVFDTCLLITAFFIYAMEYIIEYTQAFDLYVAWLTYLRFAFALSHISQTGSVYITVTVTIERLAVCHPRRSKALCSSGGAAWSILGVTAFAVLFNATKFFELEVTVNPECPDGSNWQSYILLPSRMAANSIYQQVYALWLTNIVMVFLPFLTLLVLNSIIAYTIKKSLENFDCHHQRQVVTGRNELKEKREATLVLVIIVCIFLICNFWGFVLTLLERIVDYETLVVKHHAFYTFSREAINFLAIINSSINFVIYIVFGKEFRKELIVVYGCGMRGITMRLPVHDKFSIWRTWTRRSKEIRHSLHRSKMESQRYSFSNFNCAELEHLEQANFDEESSADCRTNMKNTKTLPAKMLQNGRPVVDARYDTAKKKTTLTLVTGEHREPKKQKVCSTTEGTSC
>Hba_08923 transcript=Hba_08923 gene=Hba_08923
MWTFLTFFTPQLFDFAYWSRLPLIFLLCVRVSTGIGQGDAIPLLTKIKYLLRCFMYIFVIAFHIPSIASMVSCHLTAADKGRAFGIILAGSHFGTVIAGSIGSLLLDWLGWRILFYFVGVLSLIWYWLRVVLSQSAHYFPNVGSVDGERTLKTINKTHKVRDRTNRLQLKSCLSKPGLPATSSKVNWRILFRHPAFWLVYLLNTTFFAHYLYLGLPQLHNILVEMHTLQCSTGYLVIFMKLFQEQSYSQTYGGINACGAITGFIGVYIAGHILEATNNNWSYVFGVAGIQCIFGATMYGSLGRYIFIYKLIKFLTHPSASF
>Hba_09652 transcript=Hba_09652 gene=Hba_09652
MITFVLISQENLKLHNSYAIFFLFLKICFFNSIFIGFNIYYSDICIYRLLYTLETYFKTQDLLLDVYSIDSISFRKSPPTLFSFCSFLLHLTLIFKIDYLTYLCADLVYVILFPQLVCVVYMKNSNTGSLAGYGDYLNCLVVSSLLLIVQKKFGVIYHTIMVHRPLKFVICCTNIVCFIISLIAKIKTRKCDLENKFLVDFADDMRFYINIDFMISFVITIHKYRVFFFITKFIKKNVLTVSSPLPCISSCAAYISYFFHLYTLYLKILKKIIYMNNVSLVRSSHYFTFFIYNTSPVAVLVQYFIRIQHQIAVPFHLTSVVLGKVDF
>Hba_10668 transcript=Hba_10668 gene=Hba_10668
MAVKIILKYLLTVIDDMAEPDCKTYTERFPDPSHHPLTIVLFSVLYSIVFIISFAGNIGVIYATVRHRSLQTVQNIFIVNLAVSDVILCILSIPLTPVTHIAKQWYFGSMLCRAVGGIQAIGLFIGTSLCAIAVDRYFRLVIAPGSPLRKVNAIRITVVLWITSILVTLPYVYHMKMTYYPELKICGEFCTEKWPNVYSKRIYTLFVLIIQFVIPFTIMTICYQAVFSFLRRRAKSRLTSIAQQANLLYVVAATGADSQQHKEQLTHLIEQKKRVIKQRRRVTVILVSMVVIFGITSLPHNIVSTLMEFTDSQDILTFNGSDYMYLVNLITHLSCTIEQKKERRREREGERQEEREKERKRGREKERKKNIHTSLTTR
>Hba_12209 transcript=Hba_12209 gene=Hba_12209
MEQTQYFDQNLYENFSVSYALPLSNHDNGSLMAIAGFYALLFMLGTCGNAAIMAVVHHVKSSDARSRHNTTLTYICILCVVDFLSMLPLPMTIIDQILGFWMFGTFACKLFRLLEHIGKIFSTFILVFSIDRYCAVCHPLQLRVRNHRMVYILLGSMFLITCIMLFPILLYAHSKELIIHEKYDLSQKTITRMHLYKCVDDLGDKLFIVFTLYCFFLAYLMPLLFMIYFYYGMLVRLFKQARAVRQTLIGRRKGDEKIPIGHIAVYTLAICSFHFLCWTPYWVSVLYSLYVEIFNPNAMLETPSFKFIYFMYGVHALPYINSASNFILYGLLNRQTRGYANGFLNNQPSKDQLADSVGQISAEEESVVI
>Hba_14891 transcript=Hba_14891 gene=Hba_14891
MSICNETAKVFTTEPESISMLAPYMVSSAYILTVMVGVFGNIWVVCSIARSRKPRSLLSHSSPSDRLRAYISVLAIVDLTVLMALLVRGLYLSLPHLMLDANSCRAMFILEHSVKLASLTVLSCISIRYITIRKPFCSQVRKRFIRLTPLAALLLLTAVVATVFAQVPSVSVSSDGLNCIRSYRGRLIPRLGSYLTAVVFLANLTTISVNYGQIVRHTPVLPVVAHSRARESLVNEPRYMREMTSAIIRVGVFHVSWLPFCLLQFLPDSAIVSELTASIRMFDNFKDFSYLRWVVFIANWLTHANAAGDWIFYAAMNRDLRNLIRMYPDNLH
>Hba_14952 transcript=Hba_14952 gene=Hba_14952
MAEGKGRAFALIISIMMVVTGSLNTICAKWADSLKAEGVAFNHPFLQATCMFFGEFLCLVAFFAVYGYRRYKWNKVNISGSSGAIIEISTESEPQLPQFNPFVFLPPACCDILATSIMYIGLNLTTASFQMLRGAVIIFTGLLSVGMLGARIQPFKWFGMLFVMAGLVIVGVSDILEDDNPLDDKNAIITGNLLIIMAQIIVAIQMVYEQKYLNQYDVPALFAVGLEGMFGMIILSALMVPMYYIHVPHTFSTNEGRLEDVFFAWKEISEDPWIAVALSGTIISTKDSHILRCCFSICGADLVEDDRDSLLNEDE
>Hba_15447 transcript=Hba_15447 gene=Hba_15447
MYIDSNRNIELTSSNFFLFEIDNHTNLGINNGAERHVTDYTVMLSIDTFIPLICYTVLWYSVNLAVTTHCWTTWQEGIRRKRLVNVTTSLIHSSVSGVFLFVYFIRNTQIMFSSPMHYYSYSDSQIVLSIGYFIYDACDLLVNDRISIATCVLLFHHIASVYVLSTAIASKKFLLYAYWALLMEVSSIFLHSRSLLHISKLSATSMIAVSKAIGYMNIIAFIIFRFGVQFFLIGWAYVNMDNMHIYYVWIAMGGGIFLVINIGLFLRILHSDGLLCSSVVSHERLDALLEDNEYKSGKLEKKELLIN
>Hba_15723 transcript=Hba_15723 gene=Hba_15723
MNVSNSEENSFEPDQCDYQPQVFLEWKMIFVGYIGLITALISIIHNCLLFYAFTTSKILRRRNLTYLMWISGCDIFISICYIAIMCVQVYSDYYASFYLFYLWHCYLRFAFTVSHITLSSSSFLLMATIERYLQSTGDNRAIQLFRILDTNRTLVVLFCFIASCIFRGTVFFEVTVKYNHQCSGFSSMGLVASNLFSNPVMDAVWKFWIRKIVTVFLPFGVLAYFNAAIVMNVRRTDRDQTVKALVLFITVGSREVTRLRSRLRTVTRMLVMVVCCYLAANILDVVIAFWETIDISALNANQGIYAVTTDISSFLPILACALRLPIYIINDKQIRIEVHCKVQQVLQRLCIFCPRLARFLRVRTKWYDSKLEAERFFAASPTSFPRTDLEVKYGMGSLIMARASLSTQKELQELRKFREAVLL
>Hba_16455 transcript=Hba_16455 gene=Hba_16455
MEQNDQVKHPKSGCPAKDEQSIRSAFIKKVFMILTAMFIMLAIMCSIPFFLTSFSNWVSKNLIFLWISVCVFIGVSLTLTCCQSLRRNFPVNLIALGIFTLASGYMTMAITAAYTIESVLIVFCITACSAAITIFSVFVKKDLTSMMGIAFILSIILLFFGISASIACLAFGVTFLQTVYACLASAVMMFYLAIDVQMIVGGRKYEISPEDYVFAALQIFLDILNIFLLLLSIFGKAK
>Hba_17046 transcript=Hba_17046 gene=Hba_17046
MAEIIERCVEQIQTTTETLRRRAVAYYDGIFLVANRCSTVAERLRDVAEPIAYDIKDYIGNAMNQKSPITDIGRETKNNIVELYLGISVLLIGLSAGQLVGAFVLCHLFNIIFDRYVEFLMLLLVPVIYMNIRKNAAMDETERRTSLFGYALMIGALCGHLFGPSMTDLSPAIFFLPPLIMALLIDNELLVTPLASMDRYMFLYLGGALSVTCCTLGAVIPLGHFSLPVSFLSILHVAFMSIHFQCTKEKILNIYSQFAYVTGLLVIQLVVNATFGFNHLVKKQFPELTSEI
>Hba_18203 transcript=Hba_18203 gene=Hba_18203
MLGECSRNESYDFTRYFIIVYIGTPIAAAGVICNFILFVSRLFSRSRSRRSPTLYLLVLAILDLLMDLLYIPFFTVDALAIYNENEFLYHLWHIYAMFLFGTSRMVQFASTYVILCATMERFIVIAERSLEFLVGEKGRYGTIIVVLVAVFLLRLPAFFEYNIVYRSDCPIYLSYDYEPLLAGWEHYQIFNFYVMTVLHIFVPFAILLLLNISIVILTRRKIRGIGWAVTTFIDMPKVTELIRKESISSSKKRRELRYATWTMVSIATTYLCCSSLSLFISVMENIFTENTLLFHEDGSSTSFYTLATDVVSILVAVNSLLRIFVYMLCSLSFRPLCYASSQPPRTNP
>Hba_18427 transcript=Hba_18427 gene=Hba_18427
MTTSGNFSHICLNETDYMMHGTPLEYNLQRYVYPAIVLFGILGNIIDLTVLLNKGMRSRANTFLAALAFADIIFLTLLLPNILANYSLFTFNYYFRYFYFNTKVHLLTLANWCSAIAIWCVIAVCGDLVGIRSPLYARATWPWWRLPAVIACIVITTGVLTCYQHFEYFCLIRYFCQETQLYSRCLLVNSDIWFAKRPNPFSPAFRKFISICVMIYLFTMIIFPIILLTALNLMLLCALRKRQKHLAVGADQTERNNESQLQKTEQRVTLTVAFIVTMFTLTNGPSALIQFIRTIYQLQPQELYDVTMICSTLVICGKASNFILFCLGSKHFRVRLFKITQKKMNKKIESLSGSLLTSSLTKAIVSRRQSMPSKKSSAAGASLSKVRTHRTPVLNNL
>Hba_18736 transcript=Hba_18736 gene=Hba_18736
MLSASEIPLLLKRSHIVSGYRPLHQPRQYYYKSAFCSHNEVINVWSHFLPAICLMLFYVRPELSSESRLPVLILHTGIATLFFGSAFAHLLIYLPLLLVIVLGLQFCSTCYLFVSRPHWPRRLELRMTCLLLALWLYVPLIYRYLSTSSSDPSLHLHTRAFHWLLISGIFMGASVPERFAPGKFDIFGYGHQFFHFCIIMMAWNLCDAAHIDCTSTWSTSDTKAIACAFLLTCAFVSGTVYHLTSKAKRMKYE
>Hba_18743 transcript=Hba_18743 gene=Hba_18743
MMSYQIFFMLPPELVVAVVTIPLCTVGLLGNTCLLIATFMKKAIRSKIGRLRFAPVHDGYLVAVLATLHSICLFFELCMVKSHLRFEPSTRRECFLRIFPYTFSLLAQAIMFLVLAVDMLFAVVIPIHRLWMTLPYVSVMCSPPFAFATFAIFWSVLVMEDDFIEMCTPIQAIPPSVYWWGSYLINGINILGIFIIGTVVFALFHRDREVRSTRNSIKLTAYSEEKKVRNWERGPRRENNSHLQILRSIALLILFVCSWCISNISSHLSFRYLSKETAHYVQTYNVIIVLPTYCQSYFVTFLRSERYRSAYKQQIRWLLPGLAMDQPPIPQRSQNFRNSSIQL
>Hba_18862 transcript=Hba_18862 gene=Hba_18862
MMYSIPIAVLVMLGIKFLPPKILTSVVFVFTFIYLMIVRYLHYFFEVSELASQANGLQLLMTLRVIFYYITIAYISLKLLFLNTFLIFKIIGLSFEISDARRARTNPEDLADVKPRRYLEKDPSLFEFNYFYHFCGLFTGPYYTYQMLLDSRNSVLLTSWSPLPEIRERVLRLCWSVPLFIIFNKFFPLDNLRSDAVWEMSFIFRIIYAAAVFLVFRTRVYSAWAISESICVTLGIGIYPVESKPRTVVGPTDLKYRVELTMFVSALWHGTYAGYFMRVFHYTLVTYQ
>Hba_18878 transcript=Hba_18878 gene=Hba_18878
MNETNETTVAEHPTIGETAFYLSSGAIGSVFNALVLWIALRYIDTDDKPRQIIVINMTAADLLMCMVYMLTRPYLSYFPIFSCHPYYVTIWTVQLVSCLNLVWLNVDKLIFIQFPLHYYSIVNRKKMIVAAVTWLLLGYLAVMLDSFLTVSVGCGTSELSPYIYLPMCILYLVMILTSFIISAVIYFIAHTSTRMEARQRTKLFHRLFFLFSSTLWTFFTCLPYRALYLMHKLRKNSDSEFLDEVTNIFFRILVGTVINPLITIWTQRIYRMRLLKAFNKWQESSTSEDTAVTVNTRRPSSKAITTFEGTPFTTSL
>Hba_18906 transcript=Hba_18906 gene=Hba_18906
MNVVISLCVGQILMLPVLCKLSTGILSTPEDFAYLDRFCFQSETGHLEFTLSYPLDQPVQNLLLYFDSEDQWPRAYTQLKVPLSPYDNKLTKCIIVNDTSASTPTTWVHCSGTRTFTSMRSRWWFLASSCDLEDKGNNSNGIYVEYKLIMTNGEPTEILRFQFSDDEWLILPTDFLFLILLFILLLVNYVIGCQLTSRRLYHNTYRISTQSLLLDVTGLTMMVISYGTYAVDGVGTPFLKLMAQLFRGFAEMFFFMVLLLARGLNVTKMKLSKVDNFALFCLISFFITSYIGMLFWEIKGFDPALVYYQSESIPGYLIAAWRIVAWIFFIFNSYFTQAASPLKKKFYTSFVILLTPWFWSGPVVLCIANYVLNNWVREGVVIVNNCVVSYGYIVFLASLL
>Hba_19080 transcript=Hba_19080 gene=Hba_19080
MISSGRKCTPISIPMCMDLPYNSTVYPNLLNHQTQSDAQMAIGQFSPLVKVKCSEDIKLFLCTVYAPVCTQLEQPIQPCRDLCLSAKNGCESLMIKFGFRWPEQLDCSHFPVDGICVGENKTSSTPKSGSSISSSYLECPHSMRASSGSHFSLPLASGVLEDCSLPCAADNQVPVFFDARNRRYLRFWTGGWSVACCACAVFTILTFLIDLSRFAYPVRPILYMAVCYLVISIVYMIGVLYIIYYLENDYESSVGEDSFACGSFRSTPTLLVTQGGDNVLCSALAVAHYYFMMSSSTWWVVLCLAWFLAANLKWGAESIAGLSTYFHAACWGIPAILSVAVLVTNSIDGDVFTGLGLFLLVCGIWSMVRIRSYIKLQHADVKNISKLEKLMFRVSGFAVMYALPTAASAAILCYQAMQMPVWLDNWYTHHCLLSDRATFGFTMPRTSCHQRSETNAPGPEVFLIYAKYLSQLIVGITCAVWICSAKTVGSYQKVYSQIILRRSAVATDH
>Hba_19161 transcript=Hba_19161 gene=Hba_19161
MDIPFQSKFYRQFFIDDILDKHLCNAMDLFRPNDSFIYECRLNSTANADDLRFTIVGIFGSVVAMTSILSNVLLFYVFSTSRRLRRQNYANPVLLALFDIIVSFCYMLLLPVQVTAYRFMLVSLIKYVEFVRFGNCIQHICVTICNFLLVIASLERFFANSPDGKQKSLLVFLVHRKVAKVISIFLLAFLFKGTLLFETAIEDLPKCGELDRYVPVLINHMDPLTSARFWIRKISTVLIPFFTLFYCNCRIVMQHNKAKESQKQSLKKRSLRGSLTSSSIRKHYYEKKGVRTATKTLVLVVGCYLLSNFLSTFINFWEFSDPQKIQDNYYHYLIISDVASLLTIVGCVLRLPIYCISDVRIRKAVGRALLRCRVKALPMEVQNHLDKWSIVVVSNSL
RSNLTGMNNLFASQEGDSFMSPMDDTNHIWLMDIQEEDLPPERKPSRFRSIMADL
>Hba_20096 transcript=Hba_20096 gene=Hba_20096
MWNSSEIEDAYANMTHISASSPILAIVFGAVTTIGIAGNIALFLYIIIHKLYHNFISSHFIAHLCITNLVALILLLPNFIYTVWTGVNIFQSSNLMCRFQALMTCTVWTVMALMALCIAGVHLLTFAIHYDQLFGLRPGILCLLSWVISLMLALPSVTNGHIIIYDPSLRHCVWGHSDSGLKFLTYLLILGVLTPATFSSYAYVRVLGILYHSPIVFQSLGLYKSRFLVYAFLLSPLYQLPFYMLTMMNANHEWVDKQASWPTICVFFAFTQCLISPALYGASLFMMKEEDMALTARTHKNTPGYQHVPTHNLQAQLI
>Hba_20566 transcript=Hba_20566 gene=Hba_20566
MLDQLTLTYFVTTVRMLFIASERIALQSIAQEVAKNARKLEFVGLGLSLVSLIVSIVIFSYFRRLRVFRNMLHLHLMIAILMVVIIRLVLYIDLIFTGNHGPHQSPSDGKTINTMPFVCEAMYFLLEFKTVAFGWMFLEGFYLHNQLVLTVFNSEPRLTPYLIAGYGIPLLHTLLWLLVVLLKKDFKVERCLGSYYLEPEFWILDGPRMAELVVNLFFICNVIRVLWSKVRESHNTSELDRMKKSVKAALMLIPLGIPNIMQTIPFAPTRDNIMIFAIWTYCASFTYMYQGLMITIIYCFTNKEVNTVLKAFYSRYRLQHTSQNELRRGSRSIASHYQARNGQVTTSLDNNNGSTLSPYTQRSKKGSGDSTTKLMLSVPNCDELVNNNGYGTTSERMPLKTQCEYTEEIRITPMSNGRTVA
>Hba_20885 transcript=Hba_20885 gene=Hba_20885
MYNSKFNQLYHKYQRLFQLLNDIGRKKVFYLALVIQTLCGLLLAIAPTWWLFALLKWEQLELNQNSKNISFGLFDLVRTTNMRKRTLVCFFLWPVTTMMYYGLTMKSDLGGGSLYINFAISALMEIPIFIVYMLIDRIGRRQLVTCGLLTAGTCLIFNWIVGDNGGYYNLNNTMHQYQNVKREPVPFYWGMLQMMITKGAVTISYTAMYTYTSELFPTVIRNTAVGCCSTIARLGAISSSYIALWLVDSYGKLFVIPFSLLSVVAAILSAVFLPETMDKPMPESISEVEGTHI
>Hba_21007 transcript=Hba_21007 gene=Hba_21007
MDLVVETYGTYRREFEDTFNGLRHRGQVIQKRALQFLTDTRAQVNPQKQKLVQLVSDTSDINIPHKVVVGTFAWASVLILGASIGSFLGSFVLDLFISCFVGKVGAAVLAFLIIPIYAYNTIKKDEAGVPDAIIRFSLLFLAIIQGILAGHTISSSYLSAQPLAFFTPAVIALTFPVIHTLCIHPCLFYLLFQSIAHSIGTNRVSLFCGVLGTALTLNLLLGAVAGYLTTAYFLLTVLYISSASIVLQFVCQDVYNEVQLHTYQLALLILFVLVKNFVFLTFGSQQYVESFKKGEDLSQAIH
>Hba_21449 transcript=Hba_21449 gene=Hba_21449
MFEIFTHGVTFLNLLSILAFFTTVGLFFCGIPICRQIWKRKDTSEISGAPFLMGILGGCCWMTYGYLKNDYTVMVVTSCQVVLYSSYTIFYWIMTKDKLWISLKVGAVLSMCTILILSVHFFGKKVFPLGIICMTLNTADFAAPLAGLRVVIRRRATSTLPLPLCIANFLVSSEWFVYGLLVKDFYLITPNGIGSFLALIQLLLFVVLPRKPGQHSLLRKIFSCFKSQDECDLEDPTKEIVLDLGPDDKKITRARWSKKVISNVSTVAEELENVISKVGIHDQFAYKHKISNEYDTASEKTVETLDEKVEKIVSGDDILAQSEKEVKKLRNSLRATGESQRNMLRRTFSSPDFTE
>Hba_21467 transcript=Hba_21467 gene=Hba_21467
MTRNSYKHSIPHFTVHDRKNVGVMGEGGGDDPDVETRSAPGDLGAPPRISRTKGNEKKFSLDGSDAFHSNWRDLGKDPGHFLTRNFGGGSVMIWGAFSTMGLVDLAFTFQQDLAAIHATRSTETWLENDVATMDWPSNTPDLNPMENLWPSWLSFLGSRKWMLILLCWYCMVQGLVVNGLVPSAISSIERRYKLSTSTMGRIVQFYDFGYVLLCIPVSYFGGRHSKPAVLGVGLSLMSIGSLVFSTPHLLADSYANQSNNSLGSCALASENINGSDVISASLKSSCPAEQQNQQPGNFRFVFLFCMAHFLHGIGATPLFTIGVSYIDENVGPALSSLFVGIFYSFGVFGPAAGFLTAGTLLRYHTDFWHLPAEQILRVSGDTDPSWVGAWWLGFVMASFIALLAVFPIICLPKVLPESLKWHRTRLRDATQSGRRRTPECCGLSGTNKTAALNSDTPMQEHASLLYSSIPARGRGPLWYKLWLDVRHIPIAIYRILTNAPFMIITIAMIDIKLFYLFDINNIK
>Hba_21609 transcript=Hba_21609 gene=Hba_21609
MPGIHTFYDGSTVMQPISNLIGVEVDKVNFVLCMFSSLPLAFIYNKFMQANQVSRQIRTLFPLLIGLAFCFFCFGRAVKHLLANCLVSYALMYLAPSKHVHILVFIFSMGYLMVIHFYRWLILSAYYDITGPIMVAVQKITTLAFSLHDGRARKKEDLTELQKREAIKYLTLPYFQWFMWWFIVILLIRINYYFAWTFADAVCNMSGFGFSGFDENGSAKWELCTNVKPYQVEMAQSFKETLDGWNIQTGGWLRIAYDRTPISMRTIATYVLSATWHGVSIGYYITFFTGALFTLAAQTFRRCMRWRFVDFPKYKLVYDFVTFFSTKVALAYATYAFVTMDWDPAITVYKRVYFVVHILAIAILYVLPIFCHPQKKGITVAEPEKNRWTMVSETDVVLKKNV
>Hba_06453 transcript=Hba_06453 gene=Hba_06453
MADEISSVSSSSKSEMDFKQEMPNIEMPPSVLVLGMAGSGKTSFVQRLTAYLHARKTPPYVINLDPAVATVPYPANIDIRDTVKYKQVMKEYGLGPNGAIITSLNLMCTRFDQVLNMIRKRANQYSILIDTPGQIEAFTWSASGSIITDSLASSHPTVIAYIVDSARATNPTTFMSNMLYACSILYRAKLPFIIVFNKVLFYYYFFIITYSFILTYSVLSLLILSSQTFFLVSVSSTTGEGMDHFMIAVEESVEYKKDYLPMYKRILEEKQQLDMEEMKRKHDEVSSDLEVLSLAPSDNIAVNKIHLGGVAEENAEDANTLRFYYAEMEYNIYDINVLGTGLRQWSFKLKGLVAKLEFMKKIPSWFDKIGSNVKFMRSEPVFVIGCTGTGKSDLGVAIAKKYNGEVISADSMQVYKGLDIATNKITDKEAMGVTHHLMSFLNPSSSGYHVHHFREHGLDVIDVIRSRGHIPVIVGGTTYYVESLMFQDNFINTPRDEAHVFADLDALSAQLYQLLKENDPESASLVHPHNRARVRRAIEVFRLTGKKKSDHLREQKQNNGGKEFGGQLRFKDSLIISLDASLEVLDQRLDVRVDKMKNMGLRRELELYYDENKSQLENGSFGVLQCIGLKEFIPYRLSLEERQTLVGDKLFEEGCEEVKLHTRQYSRKQRNWVNSRLVRRRHHGELPSFKKLDTSDVHTFLSNGIKIVDDWMSGEEFREEELSTGLSYVYALSSVHYQYLCDAYFSYDGSYLERHNDSLVDESTKVAALKKVTISIGDDVREQNRLLQEMDGEFNSSKGLLMSTMKKLGIVSNAGGKNMLCYLVMFAMFVFFIIYYLARIVLCHRQMRTVTNYYLLNLAVADASISIFNTGFSWSYNFYYIWKLGSFYCFNNMMGITPICASVFTMIVMSIDRYWAIVHPLRRRPGRKATVAVICLIWVLAIVCGIPAFLTSKLELNYFFDGDDLFPDTLCLTDNYPDGTSQTSLLATLYNNGLITVQYLLPLMIVSCAYYRVGVVRKDKAVGDLRHAKSIAAKKKAAFMLALVVFIFMIVWLPYNAYFLLSSVLPLPESRKVILYNLHSRQICNNQIVMNPKNKFRVGFRYAFRWLPWMHSNRGEYISILSSQVRPSLIPTQTAATEF
>Hba_06469 transcript=Hba_06469 gene=Hba_06469
MGEPDHVPENNIDVDSPAEVLRQRVANNDSLPRVSSGRRVSSVELVRGAPPPTKKASVVIFETPDSPPAVRKPASWWRSLIMDDEKTAAPPEIIISPEQMNGVALDERNRNMSTASMGDRELFSWGGKDAGHHNLALFEEPSMPFFSSYLKAHTTPGPLERMQSENQKKKADLGVMLGVYLPTIQHILGVTMFIRLFWVVGIAGLGQTFLLLFLCCLCTFLTCISISAVATNGVVESGGAYFMISRNLGPEFGSVGLLFYLANTVATSMYLVGGVEILLLYIFPGLTIGGTEVHHDTDTFGMMTNNLRFYSTILLLVEFLIVAMGVKFVQMLAPVRIFRAAVTFFKTIAVSLVCVILSILACYAGGVEKTLDPLSGQRYIHGMKTTSPLICCTLFLFRVCMFGERLLQSKFVIPNNGSIYDMCDYCNLNNTFLLENLCPGKNCTYDTLSTTVSCINGFPGFKSSALVDNIGSQYVDKLYSTVDLKADVTRDVFQVFSHYSIFIPFLLMLYSTLRYFS
>Hba_08130 transcript=Hba_08130 gene=Hba_08130
MFHSSDSILFFLLGSAASLFSIFTIFSNLFTIFLLVRYRPMQKSISNLYILSLSIADVLIGLTVFVFFAVHLTSTSTFWFPERSWLCDAWQVADFVLSTVSLYSICAIALDRVWNLEKPLRVFKRSRLAIKLILCIWISPFVIWIPYYYFVTESRMLGSESMNHTCTSEYRLPFAVPFIAAPILYIPAIALVAMFVRISVVVHGHLSFLKEHSNHPGTSLVNTNSFRGSKESTPFTRTPEQTKKFSEGSQTSGDGYGTIRTPRSQSPRYLRVELTPLSEEKSSGRERSLSEQLTGRIEQFDVVRAAKAVALILICFLVCWLPFLVIWPIKLYCRSCIPNWLYIVSIFLNYLCSALNPLLYTLSSPRIRIVMQSYCSFFCATKRRMKHSYIVRSGTMYYALWRVFV
>Hba_09338 transcript=Hba_09338 gene=Hba_09338
MSLSSIKEQNVIILGNNHPAAGPDLTRDLARSIPSSDAIITENDASWQPKVLIDPEGRQFIAPLKIKTIMREKEFRKEAAEIAEVLQHHPVQSTVPLSIWLWSLFGCSLVVSCGIIPAFLLPENSSELHTLEGKRNLDLLLSFAVGSLLGDVFLNLLPETWSSCNVPTEKIGVWTIAGLLICIIVEKLCATTKDGQYRVCAIMNLVANVMDNFTHGLAVGASFLVSPKFGLMTTFAILVHEIPHEVSDFAILLRDFNRNQAVKAQLVTAFGGVLGACVALWIHTESFAAASWVLPFTAGGFINIALAQILPELNGEIDRRLQRVKIFNLFYRQNTKQLIMIGLGVSIMGLLNQTSFSGKNSIEKQSRPFLPGENYMNKDNNFPNLPFSTAANFRATFSLYELVDIRLFAFITPLYFLWFELIMKYFIKLLKFKIHQQKTPSWKSLMRLDATGSHANMKK
>Hba_09978 transcript=Hba_09978 gene=Hba_09978
MSDGVDCSFELSAYNALLSLLFIVVFFLSLIGNMVWSKIFYINKVNFNFRSVACIPVTLLSELTHCWLLGRFMCKMKVNSWRFKDVASAVGVCASAYTLAVIAVERYYAICRPLESRKWQTKKRALIISFVWFFSFMANVGSLAIFDSVPYRAQWTCDTIRGPIVDFLYQLYVTFVLLFIPLSLMVGLYGHVIFALTSAIGTNNFIEQAILGNDLPNRTSLSDWLFSTVSRAPSVKKCSTGEKQSLTIPASQSVSRHSRMGSITILFGTPRSSFDSSMLLRSTHQDKILVAKRKVTRMLIIIVIVFAACWIPSYTWWLLVRTADLLFHEEQRLSLEKMKQQQCSHGNKTIIFSRKEFLSYETAPTLALIHEEIFEGLDLWHGLNMALTILTYVSSMTNPITYCFMNKSFRASIFSYCHPRKSKKRRRIDDFNLIISLLYELNSTKTKYNFFFK
>Hba_09980 transcript=Hba_09980 gene=Hba_09980
MKRRLCIGIALIGGSRFIIMDEPTAGVDESSFDYRSTVTRIIAEVNIPLKLSDETDEELVFSITPHREYIVPKRKMGFLRRIKKSLEKRIDMNTEQVVLMNDRGRNTSDNSDTKSIGPEGKSSKLVQAIMLPIALLFACELYSKIQNIDSQVMLTISQSPLPLVQSLFGNDTDSYVRCVSGVPSLRDELSMIWAKMRKEDRPLRVKTMVTASGCAYGSSSGTFNFDFNRSIAYNADLICDCMDNLGWNCTQENYLPSLSSFTLNTTERVWDLSFRNISQMRMATRNSVNKTSDSPFFMGGFSLGHKNFRARNSDQINTAKKGWIELLEGKFFLYSVILNLFVLYLFSLILSVFYRQKLLVFRITIIVLALCIIPASFTVFLEDRVSDSYHLQIVSGLSKNIYWLTGYLFDMKTIIILQTVYTLSIILILLIYIMLGVKEITYSVATFLSFFLLFWIYGYIYIYIYKKAFSATLWNYILQRFFRVPALSFVMIAMGTFFLGIVGSLTIIIEEMMKRDPTLKTSHTICSYLFLMLPQYNLGMAMFRGSFVYQLVQIGESYLTDLNRPDLVHALPIPYVLEWDLMGIHCIFLLFHILFAAIILMLVEHKSIGFLSLNNIEFTSNYRRKEIEYTKRLLEDSKEEDVDVIKEQRRIEAIENMEGAPLIVKRLAKAYSPSHLFLSQGNIFIENIYTNIAVVCLLNKDKLRNFGVTRRLFSICPSGIFWMNHLQVWTPDLNNSFGKLLIAFEEAENRWYVYLSHQISLYKFFILQLKTTMSIEDFSLSQSTLNDVFQSLSSKKNFSCEKKIIATSSNNLKMISQTYQIFHFHL
>Hba_12258 transcript=Hba_12258 gene=Hba_12258
MDLLNEVDYNDADNSSLPAGGTSTVQPAQEMCYNDHAFMIFMYTTDDLLLAASMAATVFNVMVIFCAVKLFKRSGDTMHLFILNMTIGDLILTVFCHPNELLIRKHDFLQQIHLCAVVHYFNWIGLASGLSLTMLNIDKLIYFRWPLNYDRSMSKRRAALFCLLIWGVSVGYMSPGKKFYYEIFLILFCVLPVTSSLIVSIYLFNLTRQKRNSASGGDSSAFKNKSSGDREICLQPWEWTAMRVPLEVTEDLSNVKSLVFIFATTAWTSCSLLPYRIMNLARIHLFTWSELTCEGKQMMNWVAWILIYLLTINPVRVMPIVNPLITSLIYAPYRMTIKKFLVNIPVGNRPFYSYQGENIHSDISYQFVEIEIVDHRPLNWPCDNPPKYVLLKFISIILIQTPILTADVRMSMGDSPWTPAKSPSKERNQQQMRDNQKE
>Hba_13770 transcript=Hba_13770 gene=Hba_13770
MRQSDETQELSLIFPISSYPVTTIQDWSNKHLFTSSLDYWNQEQCSIEGNIVENRIYGTMEDTPKDCTISSDDICLANDVASVDPHVNKLIEQRIEILTQFTYTSFIDKPAWMMLVSAVLLACAFLDLVVVSQLSFANAISHLAINVIIMIYCISQINNWSLSSPKEFKRMLKWSHVAAAFFKAIFGLCGFLTFGELTQVLVNIVLVIKALLSYPLPFYAAVQLLKDNLFRGIANTPFTGCYSPDGSLREWALFRIVLLLFTLFIALSVPYLIELMGLIGNITGTMLSFIWPALFHLHIKGKKMVDRDKKFDQFIIGMGCTICISGVYFSLMELIRAVQENER
>Hba_14446 transcript=Hba_14446 gene=Hba_14446
MVEFEIYSPRYSKVMSAVDMENCSPQSDGILRCPQPGTPPIEYYVRNVYPPIQIWEQNSTEIRRRCASSAPSNYLIVVYRYRRKPWGIDIELQPKVLLVVVIFVMCFVVGICGNSSILTIIRGVMEDRTRSRRSGDNAILYIAALCVCDFLMSLSLPPAILDSVIGFWIFGTWMCKLHHICGSVGRIVSTFLITAMSFDRYVAVCHPHHHRLRSRSFVLGMIACLSIFAFFLLLPMLTYARAKEMVLHELKAIDFNITRVRVFKCSDMMPPPIFYWFTSTTFILGYIVPLVLIVYFNLKLIKKLYAHKRFSVLYAIVMSIVGLPTTTSELLLFVIYCVHLLPYFGSSSNWILYGLLNTQLQVSVMKNENQLNDEHMSIITANTNNHAELRNRVLERATRSIGDAGRSFLDAQMNNVVQYAACNGAALIQSESETSMHFYQDTEQTHL
>Hba_15196 transcript=Hba_15196 gene=Hba_15196
MGKELRLLASGGKSVEVQGCPYSFVNQNILYVSSYLLNPITIASTAILSFTLVNNLIVASFVFFFFRGNLYPCISLLAVSAHLSLYTVIGVASLFLRFPSNLQRFISILGLLVGFLTLVTGNWLLNGDWSFISDTYGFMKDSHLHLTVTLLLVAVFSSYPTLNDSSIYLSLLPIFDQYKKYVLFILFSTNNVVILDPRFTLVIGGTIVTCVILMPIMWHMWIVAGSGNANFYFAVTLIYNVSQIYLMMDLMFAYRKEADDICANLVTPKTNFILY
>Hba_17037 transcript=Hba_17037 gene=Hba_17037
MFEYVLSIAYCESSSTSKGEMEQLVEFSGQILLTAAKQLDFDHVLAEVGDFGAYQIIFFFVICLPASLPSAFSAFNQPFVVGNPPHKCRVPHGREDLIPITTDKSILSCFQYNQSQVDSYRLFTSIPDAYRDFISLVPCQMGWEYDNTTYIDSLVTELMRIARINGKRDIDVDDMLAVLKANQNSTEEEQKKGHNVTDLFKTENLRKKTIIVTYIWVMNAIIYNGLTLNVSNLPVDDYWSFIINGAVELPAYFIWPLLNVVGRRWTLAATMIVCGIGCVSAMFMPKDYPWLVASASFIGKFGVGSGFAVIYIFAGELYPTVVRAIGMGMSSMVAGSGLLLAPHIVKLGDYMKILPLLIMGLMALSAGICTFFLPETLGAPLMTIEDAENFGKKSKQGMFSGARNRKRESAPLLTPLPKRKAGSIVV
>Hba_17528 transcript=Hba_17528 gene=Hba_17528
MLSIKFCLGLLLGAVSFCGLLFNIAVLFAVLLIVVRHQKSPIFIISASNIFIDSLQLILAVGYLTPSIISNQWLFPGGRSDPILVFMGGVFLMLWYNGSATQILVAVNRFVVVCMPHIHFFCTRNVSLVTMIYPLSMILAWISQYVSPCCQFSFDHTFLSYSYTLLNNTNNYSNTYIDLPLNSLSSGICVFSYAYIIYNVRVQNKIVDVALTDAGRHKRRMKEYTLILVTTVRFKMIAHFQVADSSGRPSKLNDEKREILRIASNTISRLLIEMVVVLIISYLLLLALQFVESHKSRCNYKRSLRKQFEQEEPPEAASSTLFSTRSNKGRMQLKHANQYAYIPFDVLQLVILMSVTDNALGCHEPRRLMF
>Hba_18892 transcript=Hba_18892 gene=Hba_18892
MSFMRLPGIVLVIFASGTQRNLRIFLRQHTRRTITLGNTVDDQQLGVSWTAGALISISLAGFLNFIDPELGQVSKTVHGHNKPITAITLSGDKKWLFTSDFEGHISKNFILCVLVFIIIVNFLSSCFRTRCCSNDKFSRTRSNKPRRPNSFQTASRPVSPMHFRTTDELEEEEDKSSLASFSSHYESKMSSPPLPHRRAGVIMGVRALGCPQHKNMCVLSCPSRGSYPKMKEYEPPKLMLELKPNLLERESSSRSDSIIKDMPTLEKEEQKDKAEFSKYASVKLRQRAEVPHLLNLQDDEQTPKDVYPRKAEPLSAINNCFDMSALDFDLFVLDAPNRLFYLSIFDLKEMGLLQIEYVAEDDTLADISLKEATSASLRTSNIILGTLFRFLTFYIYCFKSESFSKKLRESLIYIMYAHISLVNIIDLMFTVLISLIFVANGAWVLGVTWCHINATMQEFCQLYTLLSIMLMAVERAVGVVTGTAALLPNQRGIDDAKLLSGKRIAVLSSLAVVATAISLPIVTTVIPVKEYKNRYLFFTVKTLEKIIYRRLMSGNYSGAPLGYPIARMVIYCGSLCVLLICIGAILRKREMYSLPSQSHEYSDFIKRNRAMQDHISRAKLVCFSI
>Hba_10155 transcript=Hba_10155 gene=Hba_10155
MNETAESIRFRSNIGTDIVIANLISYTIELFVLILSIALTLLHCIISKLGSRFHPMLRTLLVFMSVGMLGIAVSRIITVPIRLNFGHTTSVSWMSTLDTVVINSHEVSIVLVNLAVFLIAIERMTIFLGSFFLCYKYRSDSVEMNVSYILTTTNLTGLMVNFLRKYVCVIGIISCFQIILWSNRRSQMYLINSNRALSTSYQCRENQSVVGLIVPVYFLLFVVRLCQFIFRIYSFSVGFALGALPVRILGHFFNSKLRLKICSKIVKNQIFNKKQKLNFDLSQKAEKRYLFVATENVHHATASYTTLVHHTHCRLAHFPIHHFY
>Hba_12148 transcript=Hba_12148 gene=Hba_12148
MTNNQSGNGNLTTDDIDNKDEKEEAVIEIGGLVRPPMNQDEFDKRFFYKQPEEKSEFSRQFRKFVRSYYEPFTSLTLFKYSLFSFIPIIDWLPDYKWREDLLFDMIGGLTVGIMHVPQGSFAVVSLMGIAVDRITNVSYLKELEESGSNITLTTNDLPTPIEVASVLTLTVGVIQFLMGFFRLSFLTTYFSDQVVAGFTTGACCHVFVSQLKDLLGLRGMPKRSGPGYMLMKLYDIALNLHRVNPASVIISVTTTVLVVGKEIINPLAKKRFKLIVPIPFELLVVILATLVTYVFQLNPLYRVKIAKSIPTGMPEPSFPRFSLIPHLIANAIGISIVVVAVHMSLAKMFGKKLNYSINPGQELYALGLSSIASGFLPVYPVCSLGRTLVNVEAGTKTQV
>Hba_13241 transcript=Hba_13241 gene=Hba_13241
MPNVDIEVIAPLVSSIFFYTIVLAVAEASRRILDQVLSKKSILYRFLIEFIGTAQICTCVFENVVIVQHYGVSSFFIITSVLGVIFGSINRGSYGTPLAPIELFHYGEITLLRFLSIVLAEICGGAIWKSAKLLWFYSLEYSDAHLQQFIASQNSCSLNYQREFLLVLAFEIIGSFAMRTVIPRLPEISGKYLAPAFISSLFSFAILFVGDSGLDPVVASSLFFGCAGLSTQWFILLYWASFIGKKYYINDEMKIFRFVR
>Hba_13307 transcript=Hba_13307 gene=Hba_13307
MNGGVALRTALAFNLTPTQDYSVYVESNTKCLQVVTSSQTSVAKHENCSFSDSLVSNASSDRKCACLTLLACVLCHLVMLLRRSLSKRLLANRMVNYRSLEMGRADALEPLRGGWNKEEFSQELLEADDLADDESLALEACREDSLYPVYFEHINAGRMQFALLFLFSLCMVETIVTGLLQEWIFLFVLIITDVIVLGITIFYKGKSQALSSVVIICSVILLGCAPFILHSSMTVLILFLCYTLLPIPFLPTVRWRVDNIYQKTAVKQRFTFKVLAAAVVSAMGLAIQIPNAEHLATDALLFFGINIVGFFVYYPTELVQRKTFRETRKCVETRMMLVRELDKQEKILLSVLPKHIAYEHKQCIQQ
>Hba_13948 transcript=Hba_13948 gene=Hba_13948
MVASEEAIRSYVSLIGGSYVTFGLLGFVCNVATVVMICTNRVYRLSAYTLMANVALADSITMLMAGVACGCDILWPDSVYPISNEKIHVLRGAQVYSDENLLISYAMLGLNRCIAICYYGTKAKSLNVSIAVFSSLTTWLIGIVAAIVGTIPEPLIGIRRDMWTISFITTDGERPTLFIVLVCLINFFGVAAQWLCSTLVLLKIRQVKQKISRNKLNQNSANRFRKQARLTFQFFYPSVLCTLSTFIYFIKPYAHFLSPWQMVLLHMILLCNHTCNPFIYAYFNDRMRMTYKEMLTCSAIRYEIRKRRTSHPFHNTRHNISR
>Hba_18568 transcript=Hba_18568 gene=Hba_18568
MDVAQPGQRSSVQITEPHLRRLVNEKSIDKVREQQIMDSILNSLIQSGATPRRARVRGIGVFGALCNRRLGRRKVGKLDPEIRDLIESGSDERPWFTWWVTVVQVVVCLVSILSYGIGPVGWDRVERDEVMDVSLTLRQVSYYEPANLWIGPKFKDLIRLGAKYSPCMRRERGLWREIQEDRSRENHTGCCVFNDRTGCYQTGQSSCPRSLATWHKWSKPLIRQQSKFDPFIQENKTERYCANPPSVDPYHWPDLTRWPVIFCSYFLNWEKYLFHTTFQICLETRTGAFLPAHMSCQITGRPCCIQLQGQCRIATKEYCSFVQGYWHENATLCSQVNCFSDVCGMLPFLGRDRPDQFYRLFIALFLHAGIIHCAITVFIQLLMRDLEKLIGWLRMAILYIGSGIGGNLASAIFVPYNPEVGPSGCQLGILAALLVDVFHHRRIIDNPSTAYRDHALVILGLFASGILPWIDNWAHLFGFIFGLLLTIITFPYLDFAPKEVNRGWLWTSTRRHVSIVVGILVVAFNF
>Hba_18935 transcript=Hba_18935 gene=Hba_18935
MHAVMILLHFRNVIMLFEKRQAKLFHLIENVYEKDEEDAGIVEEQTDRIPRELTVHMFNIFCFCFAIVTYCLDFISDIVVALSHYNENRILPAIFILILSVIPSIVLNTVSYAWICDDNYANKSSKPDEDVRSTSGNRNTRNESYLVHAIICLFQAGPLWWYYKAISYAIKFRRESDPYKQRKLFCRMVEAERDATLLRFFEAFLESAPQLLVQSYILSEYVWLRSIEGNCFHDLPKWGNLRTDTAALIIYNLFIFIKYIFIFQLIWRTLTVMSRFIVIVTFLLGYHFSIVPALIFHYLISLGHVTALQSIDSGTSSTALEMGLLLINSAIHVFMPFNMAEGSTQWRYLIAYLIEAIEGVHYKLFKHVMLRNSTNGN
>Hba_19806 transcript=Hba_19806 gene=Hba_19806
MYGLSNFTFYHCSFYIFYCLIEYLRTQHKNDVFVRYAVFQVLAKSCKPIPVLVFGVLFAGKKYYWRKYLFVIMIVIGVAMFLYKDKNTSGDKQIGLGEILLFTSLTLDGITTSIQDRINKNHRRSALMMFYMNIFSSFYLLIGLFVTGELFEFVKFVQRHPHVFFDLSLLAASSCGGQYFIFKTINEFSPLTCSIITTTRKLFTIIISVVFMNHPLTERQTWATIIVFSGLIMDAIDSKVSRSKVSTH
>Hba_20032 transcript=Hba_20032 gene=Hba_20032
MLVIGGTSVVLIFIFTELISTSFVWASEGEIVNKTDVLGEVIDANSSSIEQHGAAIVGNEAEEKKRSLAIFLIMFVIVLATLVVHMLIVFKVHYLPESLAIVILGAVVGLILSYSGRDWSEIEALSPVFFLVLLPPIIFENAYNLNKGYFFSNIWPILTFAILGTAISTFVIGLTVYILGQADLVLELSIFECFAYASMISAVDPVATLAIFQAIKVEGLLYMLVFGESMLNDAVSIVLAATAIRHSHPPLNK
>Hba_21653 transcript=Hba_21653 gene=Hba_21653
MVLSRALGLAITSGSILLFIPQILKIFNAKSAKGISLFSQILALLGAASTAAYSFSKGFVFSQWGDSFFVGIQLMIVVMQILYYSDASAYAFAFFAFCWAFTFAIIGSYIPIEVLITVQALGIPITFSKSIQAWQNYKYQSTGQLSLVSVFLQFAGTVARVFTSIQDTGDLLLIVSFGTASFLNAIIFAQFFIYWDEKKRKKMH

**Supplementary Table 2:** GPCR sequences (21) fetched from three out of four GPCR predictors used in this study

>Hba_04160
MWQGSQPQWLAKPLYGLAAPIIILITLVTNSFVVVVLSNKNLRTPTNHILLSMAITELMTGKEQEGNFNVYIYVGLHILLSANILEVKYFSGLSSCPWFLYYYTFDGLYVDHEEGLPDFWCKAHPYFVHLPTMSVININFCSLYLFHTAAIWLTVYLAIQRYIYVCLPSLVVRFCCPKRTKHIIIYICIVAFVSCVLQQSQVIGVDSWKAGLLCTRALFVHILPCALLIIFTACLFKTVNEADRKRSVHQMLSRQTIGSRCLSSNRRMHATTRMLVVVIGIFLLIEIPVALIFILHLLVVFYGLLDMSDYETINNLLIIRNFLIILTYPLNFAIYFGMSSSFKLQVGIIFL
>Hba_07805
MSTNSSNSSAECGLEPHDYLEVKFFLISVIGSLIGIFGLFGNASTALILTRPSMRNPNNLFLTALAVFDTCLLITAFFIYAMEYIIEYTQAFDLYVAWLTYLRFAFALSHISQTGSVYITVTVTIERLAVCHPRRSKALCSSGGAAWSILGVTAFAVLFNATKFFELEVTVNPECPDGSNWQSYILLPSRMAANSIYQQVYALWLTNIVMVFLPFLTLLVLNSIIAYTIKKSLENFDCHHQRQVVTGRNELKEKREATLVLVIIVCIFLICNFWGFVLTLLERIVDYETLVVKHHAFYTFSREAINFLAIINSSINFVIYIVFGKEFRKELIVVYGCGMRGITMRLPVHDKFSIWRTWTRRSKEIRHSLHRSKMESQRYSFSNFNCAELEHLEQANFDEESSADCRTNMKNTKTLPAKMLQNGRPVVDARYDTAKKKTTLTLVTGEHREPKKQKVCSTTEGTSC
>Hba_10668
MAVKIILKYLLTVIDDMAEPDCKTYTERFPDPSHHPLTIVLFSVLYSIVFIISFAGNIGVIYATVRHRSLQTVQNIFIVNLAVSDVILCILSIPLTPVTHIAKQWYFGSMLCRAVGGIQAIGLFIGTSLCAIAVDRYFRLVIAPGSPLRKVNAIRITVVLWITSILVTLPYVYHMKMTYYPELKICGEFCTEKWPNVYSKRIYTLFVLIIQFVIPFTIMTICYQAVFSFLRRRAKSRLTSIAQQANLLYVVAATGADSQQHKEQLTHLIEQKKRVIKQRRRVTVILVSMVVIFGITSLPHNIVSTLMEFTDSQDILTFNGSDYMYLVNLITHLSCTIEQKKERRREREGERQEEREKERKRGREKERKKNIHTSLTTR
>Hba_12209
MEQTQYFDQNLYENFSVSYALPLSNHDNGSLMAIAGFYALLFMLGTCGNAAIMAVVHHVKSSDARSRHNTTLTYICILCVVDFLSMLPLPMTIIDQILGFWMFGTFACKLFRLLEHIGKIFSTFILVFSIDRYCAVCHPLQLRVRNHRMVYILLGSMFLITCIMLFPILLYAHSKELIIHEKYDLSQKTITRMHLYKCVDDLGDKLFIVFTLYCFFLAYLMPLLFMIYFYYGMLVRLFKQARAVRQTLIGRRKGDEKIPIGHIAVYTLAICSFHFLCWTPYWVSVLYSLYVEIFNPNAMLETPSFKFIYFMYGVHALPYINSASNFILYGLLNRQTRGYANGFLNNQPSKDQLADSVGQISAEEESVVI
>Hba_14891
MSICNETAKVFTTEPESISMLAPYMVSSAYILTVMVGVFGNIWVVCSIARSRKPRSLLSHSSPSDRLRAYISVLAIVDLTVLMALLVRGLYLSLPHLMLDANSCRAMFILEHSVKLASLTVLSCISIRYITIRKPFCSQVRKRFIRLTPLAALLLLTAVVATVFAQVPSVSVSSDGLNCIRSYRGRLIPRLGSYLTAVVFLANLTTISVNYGQIVRHTPVLPVVAHSRARESLVNEPRYMREMTSAIIRVGVFHVSWLPFCLLQFLPDSAIVSELTASIRMFDNFKDFSYLRWVVFIANWLTHANAAGDWIFYAAMNRDLRNLIRMYPDNLH
>Hba_18427
MTTSGNFSHICLNETDYMMHGTPLEYNLQRYVYPAIVLFGILGNIIDLTVLLNKGMRSRANTFLAALAFADIIFLTLLLPNILANYSLFTFNYYFRYFYFNTKVHLLTLANWCSAIAIWCVIAVCGDLVGIRSPLYARATWPWWRLPAVIACIVITTGVLTCYQHFEYFCLIRYFCQETQLYSRCLLVNSDIWFAKRPNPFSPAFRKFISICVMIYLFTMIIFPIILLTALNLMLLCALRKRQKHLAVGADQTERNNESQLQKTEQRVTLTVAFIVTMFTLTNGPSALIQFIRTIYQLQPQELYDVTMICSTLVICGKASNFILFCLGSKHFRVRLFKITQKKMNKKIESLSGSLLTSSLTKAIVSRRQSMPSKKSSAAGASLSKVRTHRTPVLNNL
>Hba_18743
MMSYQIFFMLPPELVVAVVTIPLCTVGLLGNTCLLIATFMKKAIRSKIGRLRFAPVHDGYLVAVLATLHSICLFFELCMVKSHLRFEPSTRRECFLRIFPYTFSLLAQAIMFLVLAVDMLFAVVIPIHRLWMTLPYVSVMCSPPFAFATFAIFWSVLVMEDDFIEMCTPIQAIPPSVYWWGSYLINGINILGIFIIGTVVFALFHRDREVRSTRNSIKLTAYSEEKKVRNWERGPRRENNSHLQILRSIALLILFVCSWCISNISSHLSFRYLSKETAHYVQTYNVIIVLPTYCQSYFVTFLRSERYRSAYKQQIRWLLPGLAMDQPPIPQRSQNFRNSSIQL
>Hba_18878
MNETNETTVAEHPTIGETAFYLSSGAIGSVFNALVLWIALRYIDTDDKPRQIIVINMTAADLLMCMVYMLTRPYLSYFPIFSCHPYYVTIWTVQLVSCLNLVWLNVDKLIFIQFPLHYYSIVNRKKMIVAAVTWLLLGYLAVMLDSFLTVSVGCGTSELSPYIYLPMCILYLVMILTSFIISAVIYFIAHTSTRMEARQRTKLFHRLFFLFSSTLWTFFTCLPYRALYLMHKLRKNSDSEFLDEVTNIFFRILVGTVINPLITIWTQRIYRMRLLKAFNKWQESSTSEDTAVTVNTRRPSSKAITTFEGTPFTTSL
>Hba_18906
MNVVISLCVGQILMLPVLCKLSTGILSTPEDFAYLDRFCFQSETGHLEFTLSYPLDQPVQNLLLYFDSEDQWPRAYTQLKVPLSPYDNKLTKCIIVNDTSASTPTTWVHCSGTRTFTSMRSRWWFLASSCDLEDKGNNSNGIYVEYKLIMTNGEPTEILRFQFSDDEWLILPTDFLFLILLFILLLVNYVIGCQLTSRRLYHNTYRISTQSLLLDVTGLTMMVISYGTYAVDGVGTPFLKLMAQLFRGFAEMFFFMVLLLARGLNVTKMKLSKVDNFALFCLISFFITSYIGMLFWEIKGFDPALVYYQSESIPGYLIAAWRIVAWIFFIFNSYFTQAASPLKKKFYTSFVILLTPWFWSGPVVLCIANYVLNNWVREGVVIVNNCVVSYGYIVFLASLL
>Hba_19080
MISSGRKCTPISIPMCMDLPYNSTVYPNLLNHQTQSDAQMAIGQFSPLVKVKCSEDIKLFLCTVYAPVCTQLEQPIQPCRDLCLSAKNGCESLMIKFGFRWPEQLDCSHFPVDGICVGENKTSSTPKSGSSISSSYLECPHSMRASSGSHFSLPLASGVLEDCSLPCAADNQVPVFFDARNRRYLRFWTGGWSVACCACAVFTILTFLIDLSRFAYPVRPILYMAVCYLVISIVYMIGVLYIIYYLENDYESSVGEDSFACGSFRSTPTLLVTQGGDNVLCSALAVAHYYFMMSSSTWWVVLCLAWFLAANLKWGAESIAGLSTYFHAACWGIPAILSVAVLVTNSIDGDVFTGLGLFLLVCGIWSMVRIRSYIKLQHADVKNISKLEKLMFRVSGFAVMYALPTAASAAILCYQAMQMPVWLDNWYTHHCLLSDRATFGFTMPRTSCHQRSETNAPGPEVFLIYAKYLSQLIVGITCAVWICSAKTVGSYQKVYSQIILRRSAVATDH
>Hba_19161
MDIPFQSKFYRQFFIDDILDKHLCNAMDLFRPNDSFIYECRLNSTANADDLRFTIVGIFGSVVAMTSILSNVLLFYVFSTSRRLRRQNYANPVLLALFDIIVSFCYMLLLPVQVTAYRFMLVSLIKYVEFVRFGNCIQHICVTICNFLLVIASLERFFANSPDGKQKSLLVFLVHRKVAKVISIFLLAFLFKGTLLFETAIEDLPKCGELDRYVPVLINHMDPLTSARFWIRKISTVLIPFFTLFYCNCRIVMQHNKAKESQKQSLKKRSLRGSLTSSSIRKHYYEKKGVRTATKTLVLVVGCYLLSNFLSTFINFWEFSDPQKIQDNYYHYLIISDVASLLTIVGCVLRLPIYCISDVRIRKAVGRALLRCRVKALPMEVQNHLDKWSIVVVSNSLRSNLTGMNNLFASQEGDSFMSPMDDTNHIWLMDIQEEDLPPERKPSRFRSIMADL
>Hba_20566
MLDQLTLTYFVTTVRMLFIASERIALQSIAQEVAKNARKLEFVGLGLSLVSLIVSIVIFSYFRRLRVFRNMLHLHLMIAILMVVIIRLVLYIDLIFTGNHGPHQSPSDGKTINTMPFVCEAMYFLLEFKTVAFGWMFLEGFYLHNQLVLTVFNSEPRLTPYLIAGYGIPLLHTLLWLLVVLLKKDFKVERCLGSYYLEPEFWILDGPRMAELVVNLFFICNVIRVLWSKVRESHNTSELDRMKKSVKAALMLIPLGIPNIMQTIPFAPTRDNIMIFAIWTYCASFTYMYQGLMITIIYCFTNKEVNTVLKAFYSRYRLQHTSQNELRRGSRSIASHYQARNGQVTTSLDNNNGSTLSPYTQRSKKGSGDSTTKLMLSVPNCDELVNNNGYGTTSERMPLKTQCEYTEEIRITPMSNGRTVA
>Hba_03545
MGISIYFAMRKFISETNCLMKKFHDSLMDSFAQITRECAQRLEQDEYEEVSLSTILTIAPIFSIISFVVCIVLIVFLIFALIKRRIPSRKYSIVLSRTVADLFTSAFVGAAFLLANLHSASYAILALLYVATFGFVLISFSHLFVIILRQMSVTRPYGFQSTCTMRRICTAVAVTWIMSILYAVSFAPLTTVVLDPSRSKDVCSYGSCQKPMLVIVISFIIAFLIIVFCSYCAVTYKMKKIAHSERMHNEPEVTKKMLKFLVFGGHIALYILISILILIGAGIILHNVWLYNQIRNLMKEKCDVVEYINTLIRLETIAGGAIILWLVRIVFDVFIVFITEYRRLIPCLTTDNLEPLRNSPHSSID
>Hba_20096
MWNSSEIEDAYANMTHISASSPILAIVFGAVTTIGIAGNIALFLYIIIHKLYHNFISSHFIAHLCITNLVALILLLPNFIYTVWTGVNIFQSSNLMCRFQALMTCTVWTVMALMALCIAGVHLLTFAIHYDQLFGLRPGILCLLSWVISLMLALPSVTNGHIIIYDPSLRHCVWGHSDSGLKFLTYLLILGVLTPATFSSYAYVRVLGILYHSPIVFQSLGLYKSRFLVYAFLLSPLYQLPFYMLTMMNANHEWVDKQASWPTICVFFAFTQCLISPALYGASLFMMKEEDMALTARTHKNTPGYQHVPTHNLQAQLI
>Hba_18203
MLGECSRNESYDFTRYFIIVYIGTPIAAAGVICNFILFVSRLFSRSRSRRSPTLYLLVLAILDLLMDLLYIPFFTVDALAIYNENEFLYHLWHIYAMFLFGTSRMVQFASTYVILCATMERFIVIAERSLEFLVGEKGRYGTIIVVLVAVFLLRLPAFFEYNIVYRSDCPIYLSYDYEPLLAGWEHYQIFNFYVMTVLHIFVPFAILLLLNISIVILTRRKIRGIGWAVTTFIDMPKVTELIRKESISSSKKRRELRYATWTMVSIATTYLCCSSLSLFISVMENIFTENTLLFHEDGSSTSFYTLATDVVSILVAVNSLLRIFVYMLCSLSFRPLCYASSQPPRTNP
>Hba_12258
MDLLNEVDYNDADNSSLPAGGTSTVQPAQEMCYNDHAFMIFMYTTDDLLLAASMAATVFNVMVIFCAVKLFKRSGDTMHLFILNMTIGDLILTVFCHPNELLIRKHDFLQQIHLCAVVHYFNWIGLASGLSLTMLNIDKLIYFRWPLNYDRSMSKRRAALFCLLIWGVSVGYMSPGKKFYYEIFLILFCVLPVTSSLIVSIYLFNLTRQKRNSASGGDSSAFKNKSSGDREICLQPWEWTAMRVPLEVTEDLSNVKSLVFIFATTAWTSCSLLPYRIMNLARIHLFTWSELTCEGKQMMNWVAWILIYLLTINPVRVMPIVNPLITSLIYAPYRMTIKKFLVNIPVGNRPFYSYQGENIHSDISYQFVEIEIVDHRPLNWPCDNPPKYVLLKFISIILIQTPILTADVRMSMGDSPWTPAKSPSKERNQQQMRDNQKE
>Hba_17528
MLSIKFCLGLLLGAVSFCGLLFNIAVLFAVLLIVVRHQKSPIFIISASNIFIDSLQLILAVGYLTPSIISNQWLFPGGRSDPILVFMGGVFLMLWYNGSATQILVAVNRFVVVCMPHIHFFCTRNVSLVTMIYPLSMILAWISQYVSPCCQFSFDHTFLSYSYTLLNNTNNYSNTYIDLPLNSLSSGICVFSYAYIIYNVRVQNKIVDVALTDAGRHKRRMKEYTLILVTTVRFKMIAHFQVADSSGRPSKLNDEKREILRIASNTISRLLIEMVVVLIISYLLLLALQFVESHKSRCNYKRSLRKQFEQEEPPEAASSTLFSTRSNKGRMQLKHANQYAYIPFDVLQLVILMSVTDNALGCHEPRRLMF
>Hba_14446
MVEFEIYSPRYSKVMSAVDMENCSPQSDGILRCPQPGTPPIEYYVRNVYPPIQIWEQNSTEIRRRCASSAPSNYLIVVYRYRRKPWGIDIELQPKVLLVVVIFVMCFVVGICGNSSILTIIRGVMEDRTRSRRSGDNAILYIAALCVCDFLMSLSLPPAILDSVIGFWIFGTWMCKLHHICGSVGRIVSTFLITAMSFDRYVAVCHPHHHRLRSRSFVLGMIACLSIFAFFLLLPMLTYARAKEMVLHELKAIDFNITRVRVFKCSDMMPPPIFYWFTSTTFILGYIVPLVLIVYFNLKLIKKLYAHKRFSVLYAIVMSIVGLPTTTSELLLFVIYCVHLLPYFGSSSNWILYGLLNTQLQVSVMKNENQLNDEHMSIITANTNNHAELRNRVLERATRSIGDAGRSFLDAQMNNVVQYAACNGAALIQSESETSMHFYQDTEQTHL
>Hba_09978
MSDGVDCSFELSAYNALLSLLFIVVFFLSLIGNMVWSKIFYINKVNFNFRSVACIPVTLLSELTHCWLLGRFMCKMKVNSWRFKDVASAVGVCASAYTLAVIAVERYYAICRPLESRKWQTKKRALIISFVWFFSFMANVGSLAIFDSVPYRAQWTCDTIRGPIVDFLYQLYVTFVLLFIPLSLMVGLYGHVIFALTSAIGTNNFIEQAILGNDLPNRTSLSDWLFSTVSRAPSVKKCSTGEKQSLTIPASQSVSRHSRMGSITILFGTPRSSFDSSMLLRSTHQDKILVAKRKVTRMLIIIVIVFAACWIPSYTWWLLVRTADLLFHEEQRLSLEKMKQQQCSHGNKTIIFSRKEFLSYETAPTLALIHEEIFEGLDLWHGLNMALTILTYVSSMTNPITYCFMNKSFRASIFSYCHPRKSKKRRRIDDFNLIISLLYELNSTKTKYNFFFK
>Hba_08130
MFHSSDSILFFLLGSAASLFSIFTIFSNLFTIFLLVRYRPMQKSISNLYILSLSIADVLIGLTVFVFFAVHLTSTSTFWFPERSWLCDAWQVADFVLSTVSLYSICAIALDRVWNLEKPLRVFKRSRLAIKLILCIWISPFVIWIPYYYFVTESRMLGSESMNHTCTSEYRLPFAVPFIAAPILYIPAIALVAMFVRISVVVHGHLSFLKEHSNHPGTSLVNTNSFRGSKESTPFTRTPEQTKKFSEGSQTSGDGYGTIRTPRSQSPRYLRVELTPLSEEKSSGRERSLSEQLTGRIEQFDVVRAAKAVALILICFLVCWLPFLVIWPIKLYCRSCIPNWLYIVSIFLNYLCSALNPLLYTLSSPRIRIVMQSYCSFFCATKRRMKHSYIVRSGTMYYALWRVFV
>Hba_13948
MVASEEAIRSYVSLIGGSYVTFGLLGFVCNVATVVMICTNRVYRLSAYTLMANVALADSITMLMAGVACGCDILWPDSVYPISNEKIHVLRGAQVYSDENLLISYAMLGLNRCIAICYYGTKAKSLNVSIAVFSSLTTWLIGIVAAIVGTIPEPLIGIRRDMWTISFITTDGERPTLFIVLVCLINFFGVAAQWLCSTLVLLKIRQVKQKISRNKLNQNSANRFRKQARLTFQFFYPSVLCTLSTFIYFIKPYAHFLSPWQMVLLHMILLCNHTCNPFIYAYFNDRMRMTYKEMLTCSAIRYEIRKRRTSHPFHNTRHNISR

**Supplementary Table 3.** Pfam analysis of GPCR sequences identified in *Heterorhabditis bacteriophora* genome.

**Query**	**Pfam**	**Position (Independent E-value)**	**Description**
Hba_04160	7tm_1	33.61 (0.022)140.321(1.8e-05)	PF00001, 7-transmembrane receptor (rhodopsin family)
Hba_04160	E1-E2_ATPase	184.245(0.043)	PF00122, E1-E2 ATPase
Hba_07805	7TM_GPCR_Srw	33.339(3e-19)	PF10324, Serpentine type 7TM GPCR chemoreceptor Srw
Hba_07805	7tm_1	47.322(7.7e-17)	PF00001, 7-transmembrane receptor (rhodopsin family)
Hba_10668	7tm_1	56.312(3.3e-41)	PF00001, 7-transmembrane receptor (rhodopsin family)
Hba_10668	WBP-1	325.374(0.71)	PF11669, WW domain-binding protein 1
Hba_10668	7TM_GPCR_Srsx	50.248(0.014)	PF10320, Serpentine type 7TM GPCR chemoreceptor Srsx
Hba_10668	Exonuc_V_gamma	245.355(0.16)	PF04257, Exodeoxyribonuclease V, gamma subunit
Hba_10668	DUF5683	220.317(0.19)	PF18935, Family of unknown function (DUF5683)
Hba_12209	7tm_1	49.331(4.4e-27)	PF00001, 7-transmembrane receptor (rhodopsin family)
Hba_14891	7tm_1	40.309(4.4e-13)	PF00001, 7-transmembrane receptor (rhodopsin family)
Hba_18427	7TM_GPCR_Srw	36.336(2.7e-14)	PF10324, Serpentine type 7TM GPCR chemoreceptor Srw
Hba_18427	7tm_1	48.315(9.5e-11)	PF00001, 7-transmembrane receptor (rhodopsin family)
Hba_18427	ATP-synt_B	217.272(0.13)	PF00430, ATP synthase B/B’ CF (0)
Hba_18427	Ninjurin	215.279(0.5)	PF04923, Ninjurin
Hba_18427	WBP-1	216.280(0.21)	PF11669, WW domain-binding protein 1
Hba_18743	7TM_GPCR_Srsx	24.316(1.3e-58)	PF10320, Serpentine type 7TM GPCR chemoreceptor Srsx
Hba_18743	7tm_1	73.298(0.0011)	PF00001, 7-transmembrane receptor (rhodopsin family)
Hba_18878	7tm_1	32.264(1.2e-08)	PF00001, 7-transmembrane receptor (rhodopsin family)
Hba_18906	GpcrRhopsn4	143.398(3.6e-58)	PF10192, Rhodopsin-like GPCR transmembrane domain
Hba_19080	Frizzled	177.357(1.2e-52) 357.500(3.2e-26)	PF01534, Frizzled/Smoothened family membrane region
Hba_19080	Fz	8.112(4.5e-31)	PF01392, Fz domain
Hba_19161	7tm_1	71.322(2.9e-06)	PF00001, 7-transmembrane receptor (rhodopsin family)
Hba_20566	7tm_2	37.296(1.6e-58)	PF00002, 7-transmembrane receptor (Secretin family)
Hba_03545	7tm_1	74.250(5.4e-06)	PF00001, 7-transmembrane receptor (rhodopsin family)
Hba_03545	DUF6789	56.138(0.19)	PF20587, Family of unknown function (DUF6789)
Hba_20096	DUF5418	31.111(0.0091)	PF17439, Family of unknown function (DUF5418)
Hba_18203	7tm_1	37.322(3.1e-10)	PF00001, 7-transmembrane receptor (rhodopsin family)
Hba_12258	7tm_1	60.175(1.5e-10)	PF00001, 7-transmembrane receptor (rhodopsin family)
Hba_17528	7TM_GPCR_Srx	15.228(2.5e-14)	PF10328, Serpentine type 7TM GPCR chemoreceptor Srx
Hba_14446	7tm_1	139.311(1.3e-21)	PF00001, 7-transmembrane receptor (rhodopsin family)
Hba_09978	7tm_1	52.321(4.4e-31)	PF00001, 7-transmembrane receptor (rhodopsin family)
Hba_09978	7TM_GPCR_Srx	86.181(0.00015)	PF10328, Serpentine type 7TM GPCR chemoreceptor Srx
Hba_08130	7tm_1	28.362(1.8e-39)	PF00001, 7-transmembrane receptor (rhodopsin family)
Hba_08130	Curto_V3	338.378(0.55)	PF07436, Curtovirus V3 protein
Hba_13948	7TM_GPCR_Srt	13.304(0.0038)	PF10321, Serpentine type 7TM GPCR chemoreceptor Srt
Hba_13948	7tm_1	30.282(0.16)	PF00001, 7-transmembrane receptor (rhodopsin family)

**Supplementary Table 4.** Conserved domain analysis for the GPCR sequences identified in *Heterorhabditis bacteriophora* genome.

**Sequence**	**From**	**To**	**E-Value**	**Bitscore**	**Accession**	**Short name**	**Superfamily**
Hba_04160	13	344	3.56E-34	127.364	cd14978	7tmA_FMRFamide_R-like	cl28897
Hba_07805	25	333	1.31E-75	239.072	cd14978	7tmA_FMRFamide_R-like	cl28897
Hba_10668	40	335	1.02E-83	256.763	cd15203	7tmA_NPYR-like	cl28897
Hba_12209	34	335	3.57E-39	140.501	cd00637	7tm_classA_rhodopsin-like	cl28897
Hba_14891	27	319	1.33E-14	72.7064	cd00637	7tm_classA_rhodopsin-like	cl28897
Hba_18427	28	338	1.33E-48	166.654	cd14978	7tmA_FMRFamide_R-like	cl28897
Hba_18743	24	316	1.98E-52	173.934	cl28897	7tm_GPCRs superfamily	-
Hba_18878	17	264	9.96E-12	64.232	cd00637	7tm_classA_rhodopsin-like	cl28897
Hba_18906	142	398	2.04E-46	159.721	cl21735	Lung_7-TM_R superfamily	-
Hba_19080	177	501	NA	314.163	pfam01534	Frizzled	cl28897
Hba_19080	6	121	2.22E-68	214.968	cl02447	CRD_FZ superfamily	-
Hba_19161	55	367	1.43E-31	122.356	cd14978	7tmA_FMRFamide_R-like	cl28897
Hba_20566	34	317	NA	384.026	cl28897	7tm_GPCRs superfamily	-
Hba_03545	61	259	8.07E-06	46.898	cd00637	7tm_classA_rhodopsin-like	cl28897
Hba_20096	24	284	5.30E-14	70.7804	cd00637	7tm_classA_rhodopsin-like	cl28897
Hba_18203	17	336	9.35E-18	82.2954	cl28897	7tm_GPCRs superfamily	-
Hba_12258	48	205	6.98E-16	77.3288	cd00637	7tm_classA_rhodopsin-like	cl28897
Hba_17528	15	165	1.77E-10	60.6931	cl28897	7tm_GPCRs superfamily	-
Hba_14446	100	359	3.25E-34	128.945	cd00637	7tm_classA_rhodopsin-like	cl28897
Hba_09978	17	416	4.23E-46	161.613	cl28897	7tm_GPCRs superfamily	-
Hba_08130	12	370	6.29E-38	137.694	cl28897	7tm_GPCRs superfamily	-
Hba_13948	19	286	2.24E-12	66.158	cd00637	7tm_classA_rhodopsin-like	cl28897

**Supplementary Table 5.** The 1252 *Caenorhabditis elegans* GPCRs identified by using our methodology pipeline.

F31F4.3	AC3.1a	AC3.1b	AH6.10	AH6.14	AH6.4
AH6.6	B0034.5	B0205.2a	B0213.7	B0213.8	B0213.9
B0238.3	B0238.5a	B0238.6	B0238.8	B0244.10a	B0244.16
B0244.17	B0244.5	B0244.6	B0244.7	B0250.10	B0250.6
B0286.2a	B0304.5	B0304.7	B0304.8	B0304.9	B0334.7
B0391.4	B0432.1	B0454.2	B0454.3	B0454.4	B0495.1
B0496.5	B0547.4a	C01B4.1	C01B4.5	C01G12.7	C02A12.10
C02A12.2	C02A12.5	C02A12.6	C02A12.9	C02E7.13	C02E7.2
C02E7.3	C02E7.9	C03A7.9	C03G6.11	C03G6.3a	C03G6.7
C03G6.9	C03H5.7a	C03H5.9	C04E12.8	C04E6.2	C04E6.9
C04F2.1	C04F2.4	C04F5.4	C04F5.5	C05E4.10	C05E4.11
C05E4.13a	C05E4.2a	C05E4.2b	C05E4.4a	C05E4.6a	C05E7.4
C06A8.7	C06B3.12	C06B3.13	C06B3.14	C06B3.9	C06B8.10
C06B8.6	C06B8.9	C06C3.6	C06C6.2	C06C6.3	C06E7.7
C06H5.7	C07D10.3	C07G3.3a	C07G3.4	C07G3.5	C07G3.6
C08B6.12	C08F11.9	C09F12.3	C09G12.2	C09H5.3	C09H5.4
C09H5.5	C09H5.6	C09H5.9	C10G11.2	C10G11.3	C10G11.4
C10G8.1	C12D5.11	C12D8.12	C13B7.1	C13B7.3a	C13B7.4a
C13B7.5	C13B9.4a	C13D9.5	C13D9.6	C14A4.15	C14C11.5
C14C6.10a	C14C6.11	C14H10.4	C15A7.2	C15C8.1	C15C8.6
C15H9.3	C15H9.5	C17B7.1	C17E7.11	C17E7.2	C17F4.4
C18B10.1	C18B10.5	C18B10.8	C18B12.2	C18D4.5	C18F10.5
C18F10.6	C18F10.8	C24B5.1	C24B9.1	C24B9.12	C24B9.15
C24B9.4a	C24B9.5	C24B9.6	C24B9.7	C24B9.8	C24H11.4
C25B8.7	C27A7.7	C27D6.10	C27D6.6	C27D6.8	C27H5.6
C28C12.13	C29G2.4	C30A5.10a	C30B5.7	C30G7.5	C31A11.10
C31A11.3	C31A11.4	C31A11.6	C31A11.9	C31B8.11a	C31B8.13
C31B8.3	C31B8.6a	C32B5.2	C32H11.2	C33A12.13	C33G8.5
C34B4.3	C34B4.5	C34C6.1	C34D4.8	C35D6.1	C35D6.2
C35D6.9a	C36C5.10	C36C5.2a	C36C5.6	C36C5.7	C36C5.8
C39B5.11	C39B5.12	C39H7.5	C39H7.6	C39H7.7	C39H7.8
C41G6.10	C41G6.11	C41G6.14	C41G6.15	C41G6.3	C41G6.7
C42D4.4	C42D4.5	C42D4.9	C43D7.6	C44C3.3	C44C3.5
C44C3.9	C45B11.4	C45H4.11a	C45H4.15	C45H4.16	C45H4.8
C46E10.10	C46E10.6	C46E10.7	C47A10.10	C47A10.11	C47A10.2
C47A10.3	C47A10.6	C47A10.9	C47E8.2	C47E8.3	C48B6.5
C48C5.3	C49D10.3	C49G7.2	C50B6.10	C50B6.12	C50B6.5
C50B6.6	C50C10.1	C50C10.3	C50C10.6	C50C10.7	C50E10.10
C50E10.11	C50E10.3	C50E10.5	C50E10.6	C50E10.7	C50E10.8
C50E10.9	C50E3.7b	C50E3.9	C50H11.10	C50H11.12	C50H11.13
C50H11.14	C50H11.2	C50H11.4	C50H11.7	C50H11.9	C50H2.1b
C51E3.3	C51E3.4	C51E3.5	C51F7.2	C53A5.10	C53B7.5
C54D10.6	C54E10.4	C54F6.1	C54F6.10	C54F6.7	C55A1.12
C55A1.3	C55A1.5	C56C10.5	C56E6.1	D1046.5a	D1054.12
D1065.5a	D2023.1e	D2023.1f	D2062.2	D2062.3	D2062.8
D2062.9	DC2.1a	DC2.2	E03D2.3	E03D2.4a	E03H12.6
E04F6.1	E04F6.13	E04F6.14	F01D4.7	F07B10.2	F07B10.3
F07B10.4a	F07C3.16	F07C4.1	F07C4.13	F07C4.14	F07C4.3
F07C4.4	F07C4.5	F07C4.8	F07G11.8	F08E10.1	F08E10.3
F08E10.6	F08H9.12	F09C6.7	F09E5.4	F09F3.13	F09F3.1a
F09G2.7	F10A3.12	F10A3.13	F10A3.15	F10A3.5	F10A3.6
F10A3.8	F10A3.9	F10D2.1	F10D2.4	F10D7.1	F10G2.8
F10G7.7	F11A5.1	F11A5.2	F13A7.3	F13A7.8	F13G3.2
F14F8.1	F14F8.10	F14F8.12	F14F8.3	F14F9.1	F14F9.7
F14H3.1	F15A2.3	F15A2.4	F15A4.1	F15A4.3	F15A4.4
F15A4.7	F15B9.7c	F15E6.7a	F15H9.2	F15H9.3	F15H9.4
F16B4.10	F16C3.1	F16D3.6	F17A2.10	F17A2.11	F17A2.12
F17A2.6	F17A2.7	F17A2.8	F17A2.9	F18C5.6	F18E2.4
F18E3.1	F18E3.4b	F18E3.5	F18E3.6	F19B10.7	F19B10.8
F19G12.5	F20A1.2	F20A1.3	F20D6.12	F20E11.10	F20E11.2
F20E11.4	F20E11.6	F21A3.1	F21F8.1	F21F8.10	F21F8.9
F21H7.11	F21H7.7	F22B8.1	F22B8.3	F22B8.5a	F22E5.16
F22E5.4	F23F12.10	F25E2.1	F25E5.13	F26B1.6	F26D2.11
F26D2.4	F26D2.7	F26F12.6b	F26G5.10	F26G5.12	F26G5.2
F26G5.4	F26G5.5	F28B1.6	F28B1.8	F28D9.2a	F28H7.1
F28H7.9	F30B5.6	F30B5.8	F31D5.5	F31E9.2	F31F4.16
F31F4.18	F31F4.6	F31F4.8	F31F4.9	F32A7.7	F32D8.10
F32H5.6a	F33H1.5	F33H12.2	F33H12.4	F33H12.5	F34D6.5
F34D6.6	F35C5.2	F35E2.1	F35F10.8	F35H10.10	F36D1.2
F36D3.13	F36D3.6	F36G9.16	F36G9.2	F36G9.6	F36G9.8
F36G9.9	F36H9.6	F37B4.1	F37B4.11	F37B4.12	F37B4.13a
F37B4.3	F37B4.4	F37B4.5	F37B4.6	F38H12.1	F38H12.2
F39E9.3	F40D4.1	F40D4.11	F40D4.3	F40D4.5	F40D4.8
F40F9.4	F40G12.15	F40G12.8	F40H7.2	F40H7.4	F40H7.7
F41F3.7	F41G3.11	F41H8.2	F44F4.13	F44F4.5a	F44F4.5b
F44F4.7	F44G3.1	F44G3.11a	F44G3.5	F45E6.1	F45H11.4a
F45H11.4b	F45H11.4c	F46B6.11a	F47B8.12	F47B8.7	F47B8.9a
F47C12.10	F47C12.3	F47C12.5	F47D2.10b	F47D2.1a	F47D2.3
F47D2.4	F47D2.5	F47D2.6	F47D2.7a	F47D2.7b	F47D2.8
F47D2.9	F47G9.2	F48F5.4a	F48G7.1	F48G7.6a	F49C5.1
F49C5.2	F49C5.9	F49E11.2	F49E12.5a	F49H6.4	F52D10.4
F52D2.9	F53F1.11	F53F1.7	F53F1.8	F53F1.9	F53F4.9a
F54B8.7a	F54E2.5	F54E2.6	F54E2.9	F55B12.6	F55B12.7
F55B12.9	F55C5.9	F55D10.4	F55G11.1	F56D6.4	F57A10.1
F57A8.3a	F57G8.1	F57G8.3a	F57G8.4	F58A6.10a	F58A6.10b
F58A6.11	F58A6.6	F58E10.6	F58E2.9	F58F6.6	F58G4.2
F58G4.5	F58G4.6	F58G4.7	F59A1.12	F59A1.14	F59A1.3
F59A1.4	F59A7.3	F59B1.1a	F59B1.1b	F59B1.4	F59B1.6
F59E11.13	F59E11.14	F59E11.15	F59E11.16	H04J21.2	H04M03.8
H04M03.9	H05B21.2	H05B21.3	H06H21.2	H10D18.3	H24D24.1
H24O09.1a	H25K10.3	H27D07.3a	H27D07.4	H27D07.6	K01A6.6a
K01A6.6b	K02A2.2	K02A2.7	K02E2.1	K02E2.3	K02H11.2
K02H11.3	K02H11.7	K03B4.5	K03D3.1	K03D7.11	K03D7.2
K03D7.6	K04C1.6	K04F1.16	K04F1.5	K05D4.2	K05D4.3
K05D4.6a	K05D4.8	K06C4.17	K07E8.11a	K07E8.9	K08G2.12a
K08G2.12b	K08G2.13	K08G2.5	K08G2.8	K08G2.9	K09D9.14
K09D9.3	K09D9.6	K09D9.7	K09D9.8	K10C9.4a	K12B6.5
K12D9.4	M01B2.2	M01B2.4	M01D1.1a	M01G12.1	M01G12.13
M02H5.2	M02H5.9	M162.3a	M199.1	M7.13	R03H4.4
R03H4.9	R04B5.8	R04D3.10	R04D3.12	R04D3.6	R04D3.8
R05D8.2a	R05D8.2b	R05D8.3	R05D8.6	R05H5.6	R07B5.3a
R07B5.4	R07D5.2	R08F11.2	R08F11.5	R08H2.13	R08H2.2
R08H2.3	R08H2.4	R08H2.5	R08H2.7	R09B5.7b	R09E12.4
R09F10.6	R10D12.11	R10D12.17	R10D12.4	R10E11.7	R11D1.5
R11D1.6	R11D1.7	R11F4.2a	R11F4.2b	R12H7.4	R13D11.3
R13D11.6a	R13D7.1	R13D7.10	R13F6.3	R13H4.7a	R13H4.7b
R186.2a	R52.7	T01B11.1	T01D1.7	T01E8.7	T01G5.3
T01G5.5	T01G6.3	T02B11.5	T02B5.4	T02D1.3	T02D1.4
T03D3.11	T03D3.12	T03D3.2	T03D3.3	T03D3.4	T03D3.6
T03E6.1	T03E6.4	T03E6.5	T03E6.6	T03E6.8	T03E6.9
T03F7.2	T03F7.3	T03F7.4	T03F7.6	T04A11.7a	T04A11.8
T04B2.4	T04C12.2a	T05A7.8	T05C3.7	T05C3.8	T05E12.1
T05E12.7a	T05G11.3	T05G11.6	T05G11.7	T06C10.2	T06C12.11
T06C12.2	T06C12.3	T06E4.7	T06E6.6	T06E6.7	T06E6.8
T06G6.2	T07C12.1	T07H8.5	T08B6.3	T08B6.6	T08G3.1
T08G3.12	T08G3.3	T08G3.5	T08H10.2	T09B9.5	T09D3.1
T09D3.6	T09D3.7	T09E8.4	T09E8.5a	T09F5.3	T10C6.4
T10D4.12	T10D4.5	T10D4.8	T10H4.2	T10H9.6b	T11A5.3
T11F1.1	T11F1.5	T12A2.10	T12A2.11	T12A2.13	T13A10.12
T13A10.14	T13A10.6	T13A10.9	T13F2.5	T15B7.13	T16A9.2
T18H9.4	T19B10.10	T19C4.2	T19C4.3	T19C9.2	T19C9.3
T19C9.4	T19E7.5	T19H12.4	T19H12.5	T19H12.7	T20B3.3
T20B3.4	T20B3.5	T20C4.1a	T20D4.1	T20D4.18	T20D4.2
T21B4.12	T21B4.14	T21B4.5	T21B4.6	T21B4.7	T21B4.8
T21H3.5a	T21H8.2	T21H8.3	T21H8.4	T22B7.5	T22H6.3
T22H6.4	T23D5.10	T23D5.11	T23D5.12	T23D5.2	T23D5.6
T23D5.7	T23D5.9	T23F1.4	T24A6.10a	T24A6.13	T24A6.4
T24A6.6a	T26E4.12	T26H2.6a	T27A1.7	T27C10.1	T27C10.2
T27C4.3	T27C5.5	T27E7.8	T28A11.1	T28A11.10	T28A11.12
T28A11.15	T28A11.9	T28H11.3	W02H5.11a	W02H5.16a	W02H5.6a
W03F9.6	W04D2.2a	W05H5.1	W06D12.4	W06D12.7	W06G6.3
W06G6.6	W06G6.8	W07A8.1	W07A8.5	W07G1.6	W09D12.2
W09D12.3	W10G11.10	W10G11.9	Y102A5C.15	Y102A5C.32	Y105C5B.10
Y105C5B.6a	Y105C5B.6c	Y105C5B.8a	Y105C5B.8b	Y113G7A.1	Y113G7B.2
Y116A8C.40a	Y116A8C.40b	Y116F11B.5	Y17G9A.1	Y17G9A.5	Y17G9A.6
Y17G9A.7a	Y19D10A.15a	Y19D10A.17	Y22F5A.2	Y25C1A.10	Y25C1A.9a
Y25C1A.9b	Y26G10.1	Y26G10.2	Y26G10.4	Y27F2A.11	Y27F2A.2
Y27F2A.4	Y27F2A.7	Y32B12B.5	Y32B12B.7	Y34D9B.1a	Y34D9B.1b
Y37E11AL.6a	Y37E11AL.6b	Y37E11AL.6c	Y38C9B.2	Y38F1A.4	Y38H6C.12
Y39A3B.4	Y39C12A.6	Y39H10B.1a	Y40B10A.5a	Y40B10A.5b	Y40B10A.5c
Y40B10A.5d	Y40B10B.2b	Y40C5A.4a	Y40C5A.4b	Y40D12A.3	Y40H7A.1
Y40H7A.5	Y40H7A.6	Y41D4B.24	Y41E3.12	Y41E3.14	Y41E3.15
Y41E3.17	Y41G9A.4c	Y43B11AR.5	Y43F8C.19	Y44A6B.1	Y44A6B.2a
Y45G12A.1	Y45G12C.10	Y45G12C.14	Y45G12C.15	Y45G12C.5	Y45G12C.7
Y45G12C.8	Y46H3A.1b	Y46H3C.1a	Y46H3D.2	Y46H3D.3	Y47G7B.1
Y47G7B.3	Y49C4A.1	Y49C4A.2	Y49C4A.5	Y49C4A.6	Y49F6A.2
Y49F6A.3	Y4C6A.2a	Y4C6A.2d	Y4C6A.2e	Y4C6A.2f	Y51A2D.12
Y52E8A.5	Y54G11A.12	Y54G11A.15	Y54G2A.38	Y54G2A.77	Y54G9A.10
Y55F3C.10	Y55F3C.2	Y55F3C.8	Y57A10B.5	Y57A10C.7	Y57A10C.8
Y57G11C.30a	Y57G7A.4	Y59A8A.4	Y59A8B.3	Y60A3A.22a	Y60A3A.3
Y60A3A.4a	Y60A3A.5	Y60A3A.6	Y61B8A.1	Y61B8B.1	Y62H9A.1
Y62H9A.10a	Y62H9A.10b	Y64G10A.8	Y68A4A.3	Y68A4A.7	Y69H2.1a
Y6E2A.2	Y6E2A.6	Y70C5A.2	Y70C5C.4	Y71F9B.5a	Y71F9B.5b
Y73B6BL.10	Y73B6BL.11	Y73B6BL.39	Y73B6BL.40	Y73C8A.1b	Y73C8C.5
Y73C8C.6a	Y73C8C.9	Y75B8A.34	Y7A9C.7	Y94A7B.1	Y94A7B.4
Y94A7B.5	Y94A7B.6	Y94A7B.7	Y94A7B.9	Y97E10B.2	Y97E10B.4
Y97E10B.9	Y9C9A.10	Y9C9A.11	Y9C9A.18	Y9C9A.5	Y9C9A.6
Y9C9A.7	Y9C9A.9	ZC132.7	ZC142.1a	ZC142.2	ZC190.2
ZC190.5a	ZC190.5b	ZC196.9a	ZC204.4a	ZC204.5	ZC21.6a
ZC21.6b	ZC239.19	ZC239.9	ZC404.12	ZC404.5	ZC455.8a
ZC487.2	ZC506.4a	ZC506.4b	ZC506.4c	ZC506.4d	ZC513.11
ZC513.9	ZK1037.3	ZK1037.8	ZK105.2	ZK105.8	ZK180.1
ZK228.5	ZK228.6	ZK228.7	ZK228.8	ZK262.1	ZK262.10
ZK262.11	ZK262.6	ZK262.7	ZK285.1	ZK355.2a	ZK355.2b
ZK418.6	ZK470.14	ZK6.9	ZK697.1	ZK697.10	ZK697.11a
ZK697.12	ZK697.13a	ZK697.4	ZK697.7a	ZK757.1a	ZK863.1
AC7.1a	AC7.1b	B0286.2b	B0334.6	C01F1.4	C02D4.2e
C02H7.2	C03A7.5	C03A7.6	C03G6.16	C06B3.11	C06G4.5
C09B7.1a	C09B7.1b	C10C6.2	C15B12.5a	C15B12.5b	C15B12.5c
C15B12.5d	C15B12.5e	C15H11.2a	C16D6.2a	C16D6.2b	C16D6.2c
C16D6.2d	C17H11.1	C24A8.1	C24B9.16	C25B8.5a	C25B8.5b
C25E10.3c	C25G6.5	C26F1.6	C30F12.6	C33A12.8	C35A11.1
C35A5.7	C38C10.1	C39E6.6	C43C3.2c	C44B12.4	C44C3.1
C44C3.2	C46F4.1a	C46F4.1b	C47A10.8	C48C5.1	C49A9.7
C50F7.1b	C51E3.2	C52B11.3	C53C7.1a	C53C7.1b	C54A12.2a
C54A12.2b	C54A12.2c	C56A3.3a	C56A3.3b	C56A3.3d	D1014.2
D1069.4a	D1069.4b	E02C12.2	E02C12.3	F01E11.5c	F02E8.2a
F02E8.2b	F07B10.6	F09F3.2	F10G2.6b	F14D12.6a	F14D12.6b
F14F4.1	F14F8.11	F14F8.6	F15A8.5a	F15A8.5b	F15A8.5c
F21C10.12	F27E5.8	F31B9.1	F31F4.2	F31F4.4	F35B12.1a
F35G8.1	F36G9.1	F38B7.4	F38B7.7	F38B7.8	F38E1.8
F40A3.7	F40G12.1	F41E7.3	F41H8.1	F41H8.4	F42D1.3
F43A11.4	F43A11.6	F49A5.8	F53A9.5	F53F8.2	F54D7.3a
F54D7.3b	F54E4.2	F56B6.5a	F56B6.5b	F56B6.5c	F56B6.5d
F57A8.4	F57B7.1a	F57B7.1b	F57C9.6	F59B1.3	F59B1.7
F59B2.13a	F59B2.13b	F59C12.2a	F59C12.2b	F59D12.1	F59E11.1
H02I12.3	H06H21.1	H09F14.1a	H09F14.1b	H09F14.1d	H10E21.2
H12C20.5	H22D07.1	H23L24.4	H27D07.5	K01A12.3	K01B6.2
K02F2.6	K03H6.1	K03H6.5a	K03H6.5b	K04F1.3a	K04F1.4
K06B4.9	K06C4.8	K06C4.9	K07C6.11a	K09G1.4a	K09G1.4c
K09G1.4d	K09G1.4e	K09G1.4f	K09G1.4g	K10B4.4	K10C8.2a
K10C8.2b	K12D9.3	M01B2.7	M01B2.9	M01G12.4	M03F4.3a
M03F4.3c	R05D8.4	R05D8.5	R106.2	R10H1.2	R12C12.3
R13D11.9	R13D7.4	R13H7.1	R13H7.2a	R13H7.2b	R13H7.2c
R13H7.2d	R13H7.2e	R13H7.2f	R13H7.2g	R13H7.2h	R13H7.2i
T01C4.4	T01D3.4a	T02E9.3a	T02E9.3b	T05A1.1a	T05A1.1b
T05A1.1c	T05A12.1	T06G6.7	T07D10.2a	T07D10.2b	T07D4.1
T07F8.2	T07H8.1	T07H8.3	T10C6.3	T10E10.3a	T10E10.3b
T10E10.3c	T11F9.1a	T11F9.1b	T13A10.13	T14B1.2	T14C1.1
T14E8.3a	T14E8.3b	T15B7.12	T19F4.1a	T19F4.1b	T21B4.4a
T21B4.4b	T22G5.4	T23B3.4	T23C6.5	T26E4.14	T26E4.15
T26H5.5	T27B2.1	T27D1.3	T28H11.2	W10C4.1	Y116A8B.5
Y22D7AR.13	Y23H5B.4	Y34D9A.2	Y39A3B.5a	Y39A3B.5b	Y39A3B.5d
Y39A3B.5e	Y39A3B.5f	Y40H4A.1a	Y40H4A.1b	Y41D4A.8a	Y41D4A.8b
Y43F8A.4	Y48A6B.1	Y54G11B.1	Y54G2A.35a	Y54G2A.35b	Y55F3AM.2
Y58G8A.4a	Y58G8A.4b	Y59H11AL.1a	Y59H11AL.1b	Y62E10A.4a	Y70D2A.1
Y73B6BL.41	ZC204.15	ZC266.1	ZC374.1	ZC404.11	ZC404.13
ZC412.1a	ZC84.4	ZK1037.9	ZK455.3	ZK697.5	ZK813.5
F13D2.3	F39B2.8	K07C6.8	T14E8.3c		
